# TRPM8 is required for survival and radioresistance of glioblastoma cells

**DOI:** 10.18632/oncotarget.21436

**Published:** 2017-09-30

**Authors:** Dominik Klumpp, Stephanie C. Frank, Lukas Klumpp, Efe C. Sezgin, Marita Eckert, Lena Edalat, Martin Bastmeyer, Daniel Zips, Peter Ruth, Stephan M. Huber

**Affiliations:** ^1^ Department of Radiation Oncology, University of Tübingen, Tübingen, Germany; ^2^ Zoological Institute, Department of Cell- and Neurobiology, Karlsruhe Institute of Technology (KIT), Karlsruhe, Germany; ^3^ Institute of Functional Interfaces, Karlsruhe Institute of Technology (KIT), Eggenstein-Leopoldshafen, Germany; ^4^ Dr. Margarete Fischer-Bosch-Institute of Clinical Pharmacology, Stuttgart, Germany; ^5^ University of Tübingen, Tübingen, Germany; ^6^ Department of Pharmacology, Toxicology and Clinical Pharmacy, University of Tübingen, Tübingen, Germany

**Keywords:** glioblastoma, radioresistance, TRPM8 channels, Ca^2+^ signaling, cell migration

## Abstract

TRPM8 is a Ca^2+^-permeable nonselective cation channel belonging to the melastatin sub-group of the transient receptor potential (TRP) family. TRPM8 is aberrantly overexpressed in a variety of tumor entities including glioblastoma multiforme where it reportedly contributes to tumor invasion. The present study aimed to disclose further functions of TRPM8 in glioma biology in particular upon cell injury by ionizing radiation. To this end, TCGA data base was queried to expose the TRPM8 mRNA abundance in human glioblastoma specimens and immunoblotting was performed to analyze the TRPM8 protein abundance in primary cultures of human glioblastoma. Moreover, human glioblastoma cell lines were irradiated with 6 MV photons and TRPM8 channels were targeted pharmacologically or by RNA interference. TRPM8 abundance, Ca^2+^ signaling and resulting K^+^ channel activity, chemotaxis, cell migration, clonogenic survival, DNA repair, apoptotic cell death, and cell cycle control were determined by qRT-PCR, fura-2 Ca^2+^ imaging, patch-clamp recording, transfilter migration assay, wound healing assay, colony formation assay, immunohistology, flow cytometry, and immunoblotting. As a result, human glioblastoma upregulates TRPM8 channels to variable extent. TRPM8 inhibition or knockdown slowed down cell migration and chemotaxis, attenuated DNA repair and clonogenic survival, triggered apoptotic cell death, impaired cell cycle and radiosensitized glioblastoma cells. Mechanistically, ionizing radiation activated and upregulated TRPM8-mediated Ca^2+^ signaling that interfered with cell cycle control probably via CaMKII, cdc25C and cdc2. Combined, our data suggest that TRPM8 channels contribute to spreading, survival and radioresistance of human glioblastoma and, therefore, might represent a promising target in future anti-glioblastoma therapy.

## INTRODUCTION

Glioblastoma multiforme diffusely infiltrates the brain which confines complete surgical resection. Moreover, target volumes of adjuvant radiotherapy are limited by normal tissue toxicity and often do not include all brain-infiltrated residual glioblastoma cells. In addition to the highly migrating behavior of glioblastoma cells, high intrinsic resistance to radio- and chemotherapy, most probably accounts for therapy failure and poor prognosis of glioblastoma patients (for review see [1]).

Glioblastoma cells have been demonstrated to over-express certain ion channel types that are supposed to exert oncogenic functions (for review see [1]). Among 21 tested nonselective cation channels belonging to the transient receptor potential (TRP) super family, the melastatin family member 8 (referred to as TRPM8) showed highest upregulation in glioblastoma as compared to normal brain tissue [2]. TRPM8 has been originally identified as a cancer-associated gene in prostate carcinoma [3]. Meanwhile, there is increasing evidence that many tumor entities highly overexpress TRPM8 channels similar to glioblastoma [2]. Aberrantly expressed TRPM8 channels have been proposed to contribute to epithelial-mesenchymal-transition and metastasis of breast cancer [4], growth and metastasis of pancreatic adenocarcinoma [5], adaptation to hypoxia of prostate cancer [6], metastasis and chemotherapy resistance of osteosarcoma [7], poor prognosis of urothelial carcinoma [8], carcinogenesis of colon carcinoma [9], and migration of glioblastoma cells [10, 11]. Besides these malignancy-associated functions, TRPM8 reportedly suppresses survival of melanoma cells [12]. In addition, TRPM8 is highly upregulated in an early, differentiated stage but downregulated during malignant progression of prostate cancer [3, 13, 14] which might suggest that TRPM8 is involved in early prostate carcinogenesis but rather suppresses than promotes malignant progression. These apparently contradictory functions in tumor promotion and suppression suggest that TRPM8 may exert different functions in different tumor entities.

While in normal skin and peripheral neurons TRPM8 functions as a cold sensor (for review see [15]), the functions of TRPM8 in non-temperature-sensing organs such as normal prostate epithelium are not well defined. Reportedly, TRPM8 acts as an ionotropic testosterone receptor in the plasma membrane of prostate epithelium [16, 17] or osmosensor in peripheral neurons [18]. Similarly poorly understood are the mechanisms leading to upregulation of TRPM8 in cancer cells. In prostate cancer, TRPM8 mRNA abundance [14] and TRPM8 channel surface expression [19] are upregulated by androgens, in breast cancer by estrogens [20].

Reportedly, glioblastoma cell migration is motorized by synchronized volume increase at the lamellipodium and volume decrease at the opposite cell pole. These volume changes extrude the leading edge and retract the cell rear, thereby moving the glioblastoma cell along the cell rear-lamellipodium-axis. Ca^2+^-activated intermediate conductance (IK) and big conductance (BK) K^+^ channels exert a key role in this process pointing to a pivotal function of Ca^2+^ signaling for programming and mechanics of migration (for review see [21]). Moreover, ionizing radiation at doses used for single fractions of fractionated radiotherapy in the clinic has been shown to induce Ca^2+^ signals that are sufficient to activate BK and IK K^+^ channels and to induce migration of glioblastoma cells [22–24]. In addition, radiation-induced activation of IK K^+^ channels has been demonstrated to be required for cell cycle arrest and DNA repair since inhibition of IK K^+^ channels radiosensitizes glioblastoma cells [23]. Activation of TRPM8 by menthol has been shown to stimulate BK K^+^ channel activity and migration of glioblastoma cells [10, 11] suggesting that TRPM8 functions as Ca^2+^ entry pathway in glioblastoma cells.

The present study, therefore, aimed to define *in vitro* the functional significance of TRPM8 for the Ca^2+^ and biochemical signaling in glioblastoma cells, involved in cell migration and chemotaxis on the one hand and in the stress response to DNA damage by ionizing radiation on the other.

## RESULTS

### Glioblastoma express TRPM8

First, we confirmed expression of TRPM8 and further TRP channels in glioblastoma by querying the glioblastoma multiforme database of The-Cancer-Genome-Atlas (TCGA), and by analyzing protein expression in primary spheroid cultures of human glioblastoma specimens and mRNA expression in human glioblastoma cell lines (Supplementary Figure 1). Combined, the data suggest a highly heterogeneous TRPM8 expression in human glioblastoma specimens and established glioblastoma cell lines. Among the human glioblastoma cell lines tested, p53-mutated U251 [25] (and in few experiments also p53 wildtype U-87 MG [25]) with relatively high and p53-mutated T98G cells [25] with relatively low TRPM8 mRNA abundance (Supplementary Figure 1D) were chosen for further functional studies.

### TRPM8 induces activation of Ca^2+^-regulated K^+^ channels

TRPM8 agonists have been reported to stimulate the activity of BK Ca^2+^-regulated K^+^ channels in glioblastoma cells [10, 11]. Therefore, we determined the effect of the synthetic TRPM8 super-agonist icilin (3-(2-Hydroxyphenyl)-6-(3-nitrophenyl)-3,4-dihydropyrimidin-2(1*H*)-one) on the BK K^+^ channel activity in the plasma membrane of T98G glioblastoma cells by patch-clamp on-cell (cell-attached) recording. TRPM8 activation by icilin is supposed to depolarize and via subsequent K^+^ channel activation to re- and hyperpolarize the physiological membrane potential. In on-cell recording mode, the physiological membrane potential can not be controlled and adds up with the clamp voltage. As a consequence, absolute membrane voltages are not exactly defined. Nevertheless, on-cell recording mode is suited to analyze grossly voltage dependence or conductance of an ion channel.

**Figure 1:**
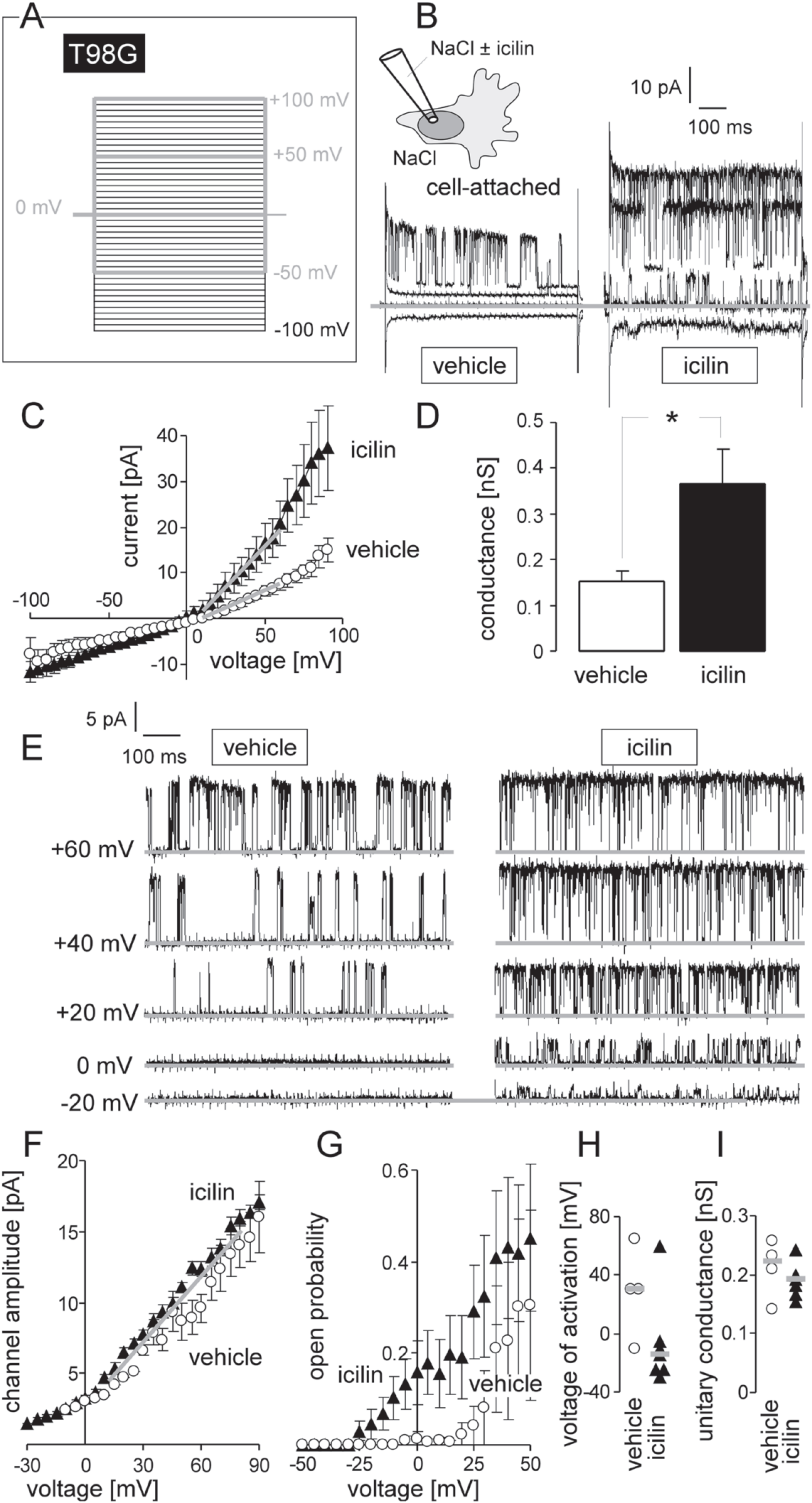
TRPM8 agonist icilin stimulates BK K^+^ channel activity in human glioblastoma cells at physiological membrane potential **(A, B)** Recording of macroscopic on-cell currents in human T98G glioblastoma cells. Depicted are the applied voltage-clamp pulse protocol (A) and tracings (B) recorded with NaCl bath and NaCl pipette solution further containing 0 μM (vehicle, left) or 10 μM (right) of the TRPM8 agonist icilin. In (B) only the currents evoked by voltage sweeps to -50, 0, +50 and +100 mV are shown. **(C)** Dependence of the mean macroscopic on-cell currents (± SE, n = 8-10) on the voltage recorded as in (A, B) from 0 μM (open circles) or 10 μM icilin-stimulated T98G cells (closed triangles). **(D)** Mean (± SE, n = 8-10) macroscopic on-cell conductance as determined for the outward currents in (C) of icilin (0 μM, open bar, or 10 μM, closed bar)-stimulated T98G cells. Conductances were calculated between +10 and +60 mV voltage as shown by the grey lines in (C). ^*^ indicates p ≤ 0.05, two-tailed Welch-corrected t-test. **(E)** Single channel current transitions recorded as in (A) from T98G glioblastoma cells with 0 μM (vehicle) or 10 μM icilin in the pipette solution. Note, that the voltage-dependent increase in open probability is shifted towards more negative potentials in the icilin-stimulated (right) as compared to the vehicle-treated cells (left). **(F, G)** Dependence of the mean (± SE) unitary current transition (F, n = 7) and open probability (P_o_, G, n = 4-7) on the voltage of the channels recorded as in (E) in the absence (open circles) or presence of icilin (closed triangle) **(H, I)** Activation voltage (H) and unitary conductance (I) of channels in vehicle- (open circles, n = 4) and icilin-treated (closed triangle, n = 7) T98G cells (given are individual recordings and the median values as grey lines).

As shown in Figure 1A-1D, the TRPM8 agonist icilin stimulated macroscopic on-cell outward currents in human T98G cells when recorded with Ca^2+^-containing NaCl solution in pipette and bath solution. This current was generated by K^+^-selective channels with a unitary conductance of about ~200 pS as deduced from the negative reversal potential and the slope of the current amplitude-voltage relationship in Figure 1E, 1F. Under control conditions, channel activity occurred only at highly depolarized voltages (Figure 1E, left) and open probability increased with increasing positive clamp-voltage (Figure 1G, open circles). Icilin induced channel activity already at 0 mV clamp-voltage, i.e., at physiological membrane potential, by changing the mean voltage of activation from +30 mV (control) to -15 mV (Figure 1E, right, and Figure 1G, 1H, closed triangles) without modulating the unitary conductance of the channel (Figure 1I). Since this K^+^ selective, voltage-dependent, and high conductance channel of T98G cells has been identified as Ca^2+^-activated and paxilline-sensitive BK K^+^ channel in our previous studies [22–24], our data confirm previous reports [10, 11] on the regulation of BK K^+^ channels by TRPM8.

### TRPM8 promotes cell migration

**Figure 2:**
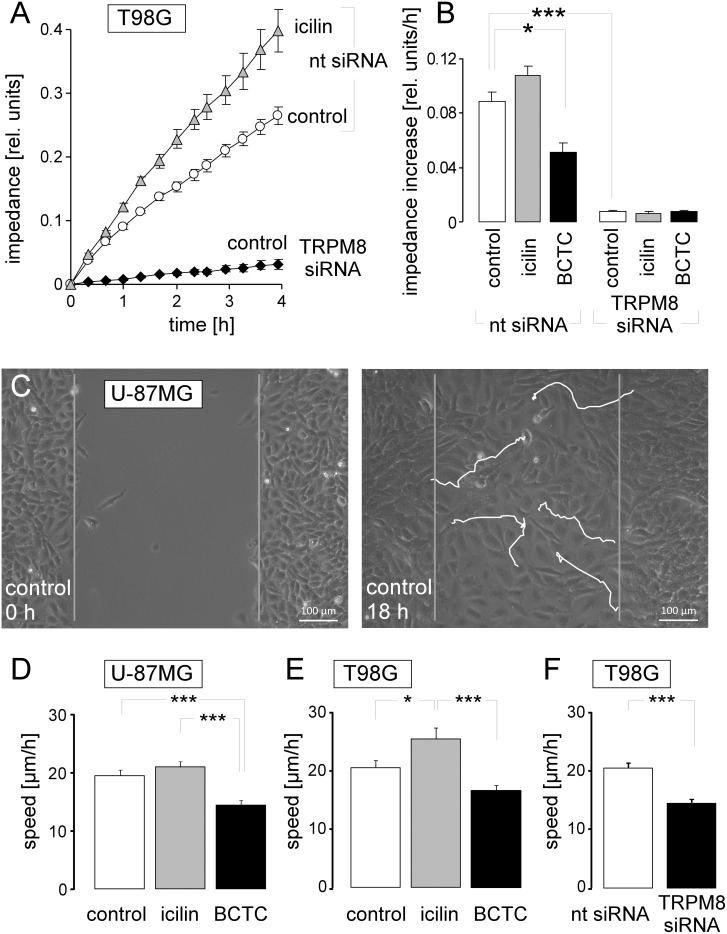
TRPM8 function is required for migration of human glioblastoma cells **(A, B)** Time dependence (A) and slope of the mean (± SE, n = 4-8) impedance (B) as real-time measure of transfilter chemotaxis in T98G cells. Transfilter migration was performed either in the absence or presence of the TRPM8 agonist icilin (10 μM) or the TRP channel inhibitor BCTC (10 μM) as well as upon transfecting the cells with nt- or TRPM8 siRNA. **(C)** Light micrographs showing wound healing of the human glioblastoma cell line U-87MG at time 0 h (left) and 18 h (right) after scratching the monolayer. The white lines indicate the migration routes of 5 selected cells as recorded by time lapse microscopy. **(D-F)** Mean (± SE) migration speed during wound healing of U-87MG (D, n = 25-26) and T98G glioblastoma cells (E, F, n = 14-45). Wound healing was performed either in the absence (open bars) or presence of the TRPM8 agonist icilin (10 μM, grey bars) or the TRP channel inhibitor BCTC (10 μM, black bars, D, E) as well as upon transfecting the cells with nt- (open bar) or TRPM8 siRNA (closed bar, F). ^*^, ^**^, and ^***^ indicate p ≤ 0.05, p ≤ 0.01, and p ≤ 0.001, ANOVA (B, D, E) or two-tailed t-test (F), respectively.

BK K^+^ channels have been demonstrated to be key regulators of glioblastoma cell migration [22] and brain infiltration [24]. Moreover, TRPM8 agonists have been reported previously to stimulate migration of glioblastoma cells [10, 11]. We tested in the glioblastoma cell lines U-87MG and T98G whether cell migration and chemotaxis depend on TRPM8 channel activity. As shown in Figure 2A, TRPM8 channel activation by icilin increased and inhibition by the TRP channel blocker BCTC (N-(4-tertiarybutylphenyl)-4-(3-cholorphyridin-2-yl)tetrahydropyrazine-1(2H)- carbox-amide) or downregulation by RNA interference decreased transfilter chemotaxis of T98G cells as induced by a fetal calf serum (FCS) gradient. Similarly, TRPM8 activation by icilin tended to increase migration speed of U-87MG and significantly accelerated migration speed of T98G cells in wound healing assays (Figure 2B-2E). Moreover, TRPM8 inhibition by BCTC significantly reduced the migration velocity of U-87MG and T98G cells. Likewise, downregulation of TRPM8 by RNA interference significantly slowed-down the migration velocity of T98G cells (Figure 2B-2E). Combined, these data clearly indicated a cell migration and chemotaxis-stimulating role of TRPM8 channels in the human glioblastoma lines T98G and U-87MG confirming earlier reports on human DBTRG glioblastoma cells [10, 11].

### TRPM8 confers radioresistance

**Figure 3:**
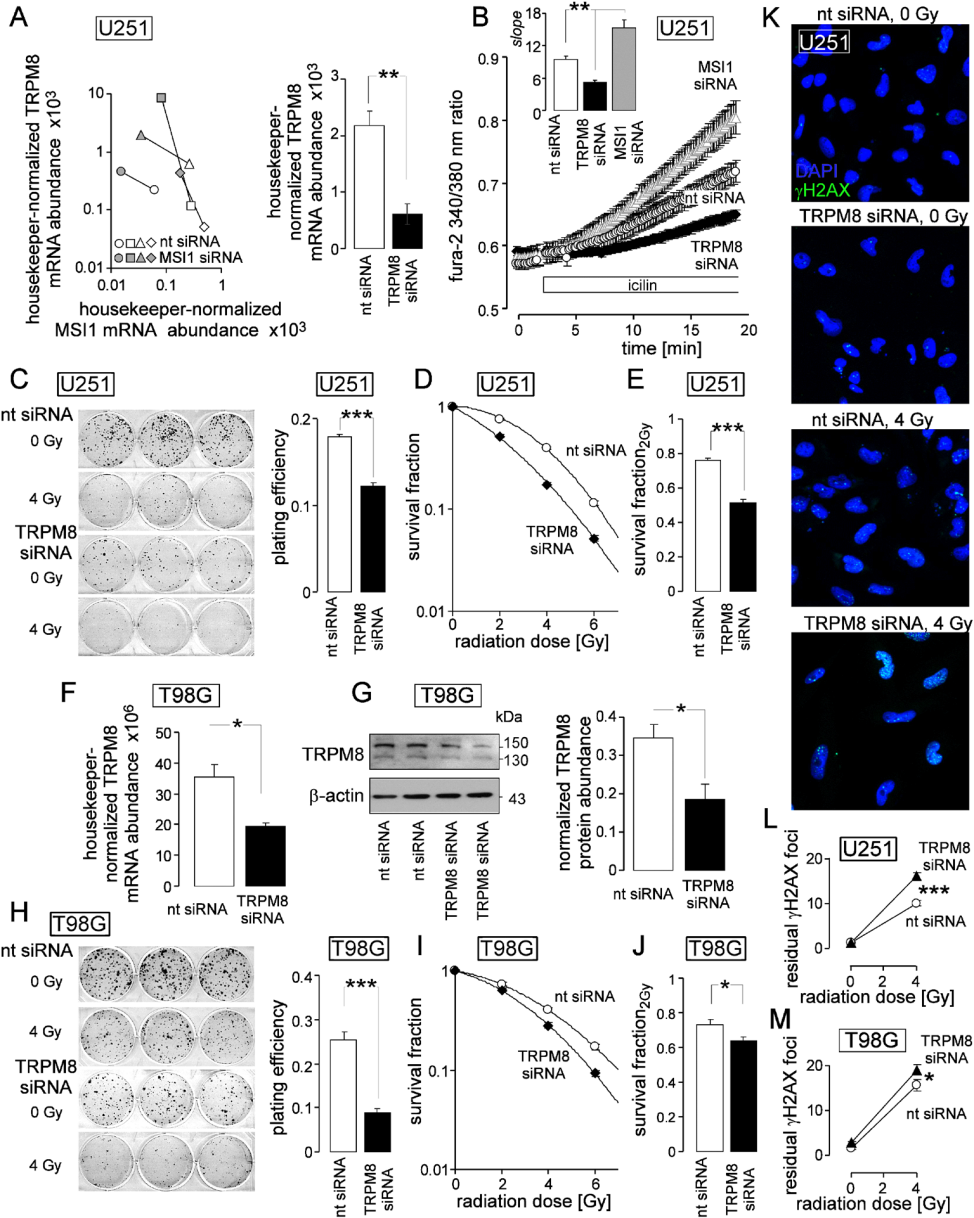
TRPM8 generates Ca^2+^ entry, increases clonogenic survival, and confers radioresistance to human glioblastoma cells **(A)** Increase and decrease in housekeeper-normalized TRPM8 and MSI1 mRNA abundance of U251 cells following transfection with MSI1- (left, 4 individual experiments are depicted) and TRPM8 siRNA (right, means ± SE, n = 4; nt siRNA: non-targeting siRNA), respectively. **(B)** MSI1- and TRPM8 knockdown increases and decreases Ca^2+^ entry stimulated by the TRPM8 agonist icilin, respectively. Given are the mean (± SE, n = 16-19) fura-2 340/380 nm fluorescence ratios recorded in nt- (white), TRPM8- (black) or MSI1 siRNA-transfected (grey) U251 glioblastoma cells before and during superfusion with the TRPM8 agonist icilin (10 μM, large plot). The insert shows the calculated slope of the fura-2 ratio/time relationship during icilin stimulation as a measure of Ca^2+^ entry. **(C-E)** Mean (± SE, n = 24-36) plating efficiency (C), radiation dose-dependent clonogenic survival (D), and survival fraction at 2 Gy (E) of nt- (white) and TRPM8 (black) siRNA-transfected U251 cells. **(F, G)** Transfection with TRPM8 siRNA effectively downregulates TRPM8 mRNA- (F) and protein abundance (G, right) in T98G cells. Given are housekeeper-normalized mean values (± SE, n = 4-8) of nt- (open bars) and TRPM8 siRNA (closed bars)-transfected T98G cells (F and G, right) as well as a Western blot of whole cell lysates of two nt- and TRPM8 siRNA-transfected T98G cells probed against TRPM8 and β-actin for loading control. **(H-J)** Mean (± SE, n = 24) plating efficiency (H), radiation dose-dependent clonogenic survival (I), and survival fraction at 2 Gy (J) of nt- (white) and TRPM8 (black) siRNA-transfected T98G cells. **(K)** TRPM8 is required for repair of DNA double strand breaks. Immunofluorescence micrographs stained against residual γH2AX foci (green) as measure of residual DNA double strand breaks in nt- and TRPM8 siRNA-transfected U251 cells 24 h after irradiation with 0 or 4 Gy. Nuclei were counterstained with DAPI (blue). **(L, M)** Mean (± SE, n = 78-108) number of residual γH2AX foci plotted against the radiation dose of nt- (open circles) and TRPM8 siRNA-transfected (closed triangles) U251 (L) and T98G cells (M). ^*^, ^**^, and ^***^ indicate p ≤ 0.05, 0.01, and 0.001, respectively, two-tailed (Welch-corrected) t-test or ANOVA, where appropriate.

To test for a radioresistance-modulating function of TRPM8, channel expression was experimentally down- and up- regulated in human U251 glioblastoma cells by transfection with siRNA specific for TRPM8 and the mRNA-binding protein MSI1 (Musashi-1), respectively. TRPM8 and MSI1 knockdown was paralleled by a marked decrease and increase in TRPM8 mRNA abundance, respectively (Figure 3A). The latter phenomenon could not be defined mechanistically. Nevertheless, MSI1 knockdown was harnessed for experimental upregulation of TRPM8 in U251 cells. The functional significance of TRPM8 upregulation and downregulation was analyzed by monitoring the cytosolic free Ca^2+^ concentration as assessed by ratiometric fura-2 Ca^2+^ imaging during stimulation with the TRPM8 agonist icilin. TRPM8 upregulation and knockdown significantly increased and decreased the icilin-induced rise in intracellular free Ca^2+^ concentration, respectively, indicating functional expression of TRPM8 in the plasma membrane of glioblastoma cells (Figure 3B).

Electro-, K^+^-, and Ca^2+^ signaling has been demonstrated to contribute to the stress response of tumors including glioblastoma upon treatment with ionizing radiation [22–24, 26]. Ionizing radiation has been demonstrated to activate TRP channels in leukemia cells [27, 28]. We, therefore, analyzed radiation-induced changes in TRPM8 activity and abundance (Supplementary Figure 2). As a result, a single dose of 2 Gy increased TRPM8 activity in T98G cells and 10 Gy total dose applied in 5 fractions stimulated TRPM8 abundance in T98G and U251 cells *in vitro* and in an orthotopic glioblastoma mouse model *in vivo* (Supplementary Figure 2) pointing to a radiation-induced upregulation of TRPM8 function in human glioblastoma.

Next, we studied whether TRPM8-mediated signaling might be involved in the stress response to ionizing radiation. Clonogenic survival of irradiated (0-6 Gy 6 MV photons) U251 cells was determined in dependence on TRPM8 expression by delayed plating colony formation. As a result, TRPM8 knockdown lowered the plating efficiency of U251 cells by 30% (Figure 3C) and decreased the survival fraction of irradiated U251 cells (Figure 3C, 3D). In particular TRPM8 knockdown decreased the survival fraction at 2 Gy (this is the clinically relevant dose applied in conventional fractionation schemes during fractionated radiotherapy) by some 30% (Figure 3E). Likewise, TRPM8 knockdown (Figure 3F, 3G) decreased plating efficiency by about 60% in T98G cells (Figure 3H) and survival fraction at 2 Gy by about 10% (Figure 3H-3J) in T98G cells.

To define whether TRPM8 knockdown-associated radiosensitization of U251 and T98G cells may result from impaired DNA repair, residual γH2AX foci as surrogate marker of residual DNA double strand breaks were analyzed 24 h after irradiation with 0 and 4 Gy by immunofluorescence microscopy (Figure 3K). As a result, knockdown of TRPM8 significantly increased residual number of γH2AX foci by some 60% (U251, Figure 3L) and 20% (T98G, Figure 3M). In summary, these data indicate functional significance of TRPM8 for DNA repair and clonogenic survival of irradiated glioblastoma cells. In accordance with the expression levels, effects of TRPM8 knockdown were higher in U251 with high TRPM8 expression than in T98G with low TRPM8 expression.

### TRPM8 is required for cell survival

**Figure 4:**
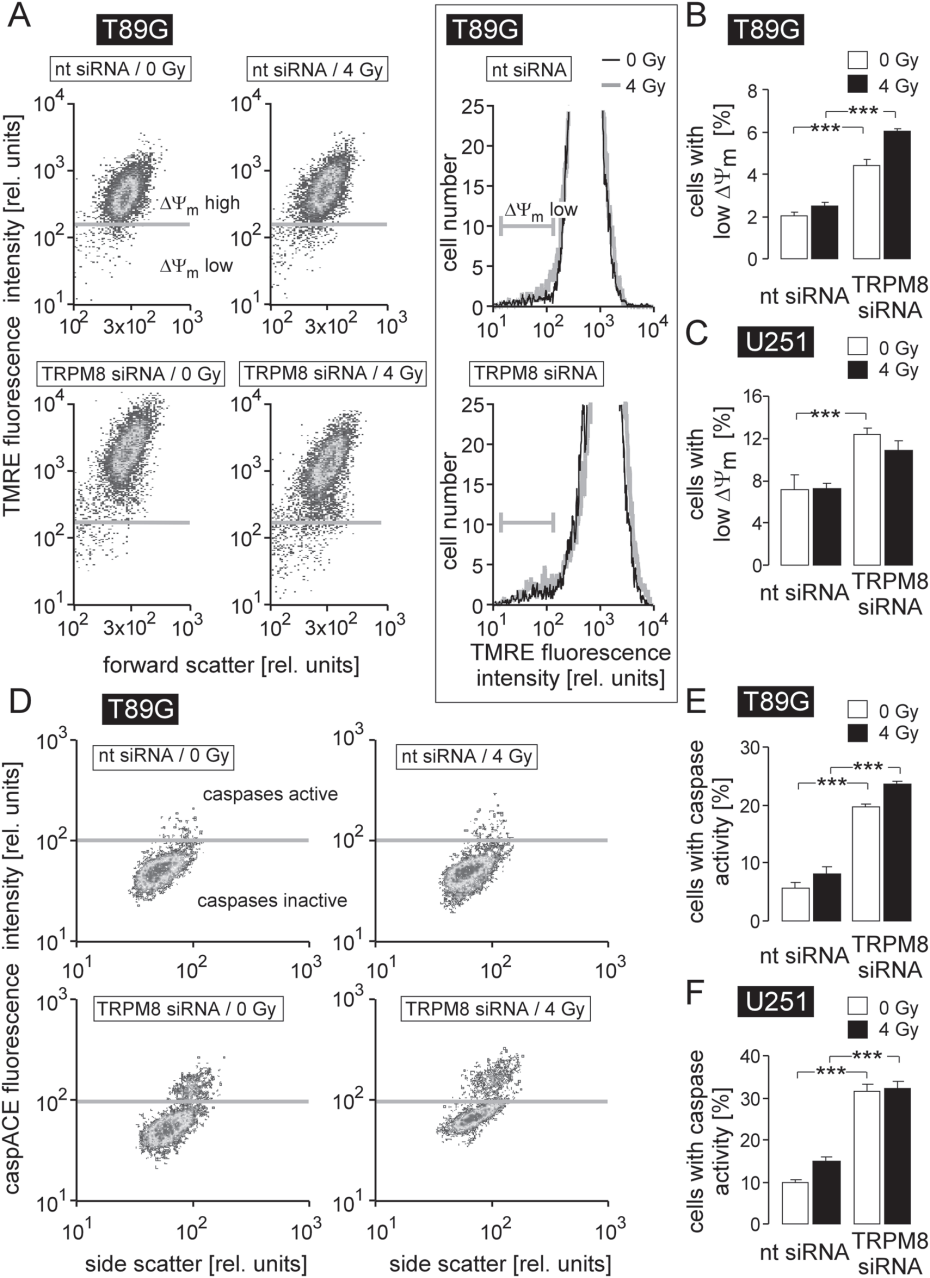
TRPM8 silencing induces breakdown of inner mitochondrial membrane potential (ΔΨm) and caspase activation in human glioblastoma cells **(A, D)** Dot blots recorded by flow cytometry showing the TMRE (A) and the caspACE (D) fluorescence intensity as a measure of ΔΨ_m_ and caspase activity, respectively, in nt- (top) or TRPM8 (bottom) siRNA-transfected T98G cells 24 h after irradiation with 0 Gy (left) or 4 Gy (right). The insert in A depicts TMRE fluorescence histograms of 0 Gy (black) and 4 Gy (grey) irrradiated nt- (top) or TRPM8 (bottom) siRNA-transfected T98G cells. **(B-C, E-F)** Mean percentage (± SE, n = 17-18) of nt- (left) or TRPM8 (right) siRNA-transfected T98G (B, E) and U251 cells (C, F) cells 24 h after irradiation with 0 Gy (open bars) or 4 Gy (closed bars) exhibiting break-down of ΔΨ_m_ (B, C) or caspase activity (E, F). ^***^ indicates p ≤ 0.001, ANOVA.

In addition to the management of genotoxic stress, TRPM8 seemed to exert survival signaling as evident from the impairment of plating efficiency in unirradiated cells by TRPM8 knockdown (see Figure 3C, 3H). To analyze possible anti-apoptotic functions of TRPM8, we compared in flow cytometry experiments the dissipation of the inner mitochondrial membrane potential (ΔΨ_m_) and caspase activity as a measure of intrinsic apoptosis triggering and execution, respectively, between control (0 Gy) or irradiated (4 Gy) nt- and TRPM8 siRNA-transfected U251 and T98G glioblastoma cells (Figure 4). As a result, TRPM8 knockdown significantly increased percentage of T98G and U251 cells with dissipated ΔΨ_m_ (Figure 4A-4C, open bars) and caspase activity (Figure 4D-4F, open bars). Ionizing radiation (4 Gy) had no or only minor additive effect on ΔΨ_m_ dissipation and caspase activation in nt siRNA-transfected or TRPM8 knockdown glioblastoma cells (compare 1^st^ with 2^nd^ bar and 3^rd^ with 4^th^ bar in Figure 4B, 4C, 4E, 4F) indicating pivotal anti-apoptotic survival signaling by TRPM8 in unirradiated cells. Moreover, the data strongly suggest that the observed radiosensitization by TRPM8 knockdown could not simply result from accelerated apoptotic death of the irradiated glioblastoma cells.

### TRPM8 signaling regulates cell cycle in unirradiated glioblastoma cells

**Figure 5:**
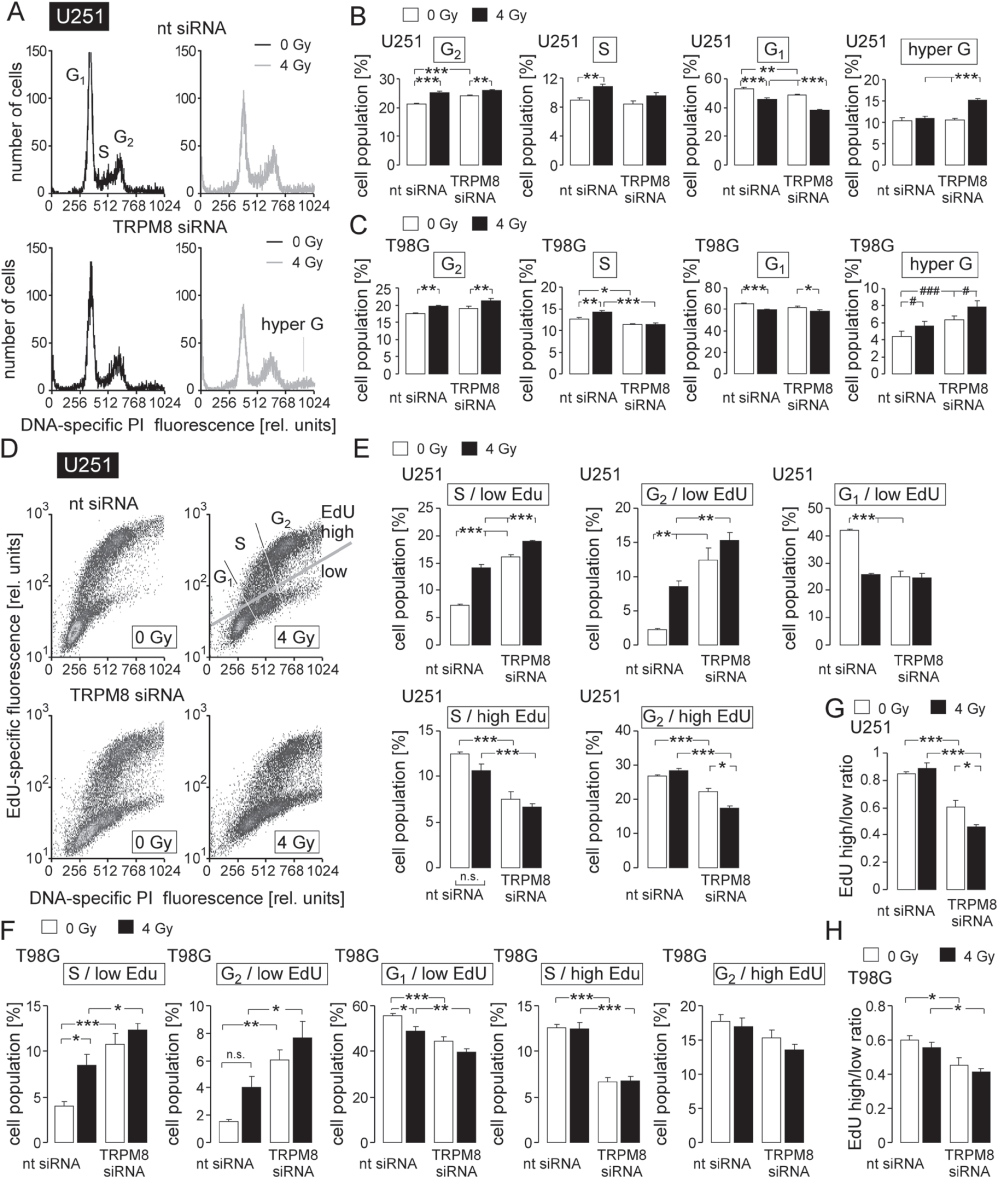
TRPM8 RNA silencing impairs cell cycle control in irradiated glioblastoma cells **(A)** Histograms showing the cellular DNA content of nt- (top) or TRPM8 (bottom) siRNA-transfected U251 cells 48 h after irradiation with 0 Gy (black) or 4 Gy (grey) as determined by flow cytometry using propidium iodide as DNA-specific fluorescence dye. **(B, C)** Mean percentage (± SE, n = 17-18) of nt- (left) or TRPM8 (right) siRNA-transfected and 0 Gy- (open bars) or 4 Gy-irradiated (closed bars) U251 (B) and T98G cells (C) belonging to the G_2_, S, G_1_, or hyper G population. **(D)** Dot blots showing 5-ethynyl-2’-deoxyuridine (EdU) incorporation by nt- (top) or TRPM8 siRNA-transfected (bottom) and irradiated (0 or 4 Gy as indicated) U251 cells. 48 h after irradiation cells were incubated for 6 h with EdU (5 μM) before co-staining with propidium iodide and analysis by flow cytometry. Gray gates show the different cell populations. **(E, F)** Mean percentage (± SE, n = 6) of nt- (left) or TRPM8 siRNA-transfected (right) and 0 Gy- (open bars) or 4 Gy-irradiated (closed bars) EdU-negative (1^st^-3^rd^ diagram) and EdU-positive (4^th^-5^th^ diagram) U251 (E) and T98G (F) cells residing in S, G_1_, or G_2_ phase of cell cycle. **(F, G, H)** Ratios between EdU-positive and EdU-negative cells in U251 (G) and T98G (H) cells in dependence on TRPM8 knockdown and irradiation. ^*^, ^**^, and ^***^ indicate p ≤ 0.05, p ≤ 0.01, and p ≤ 0.001, respectively, ANOVA. ^#^ and ^###^ indicate p ≤ 0.05 and p ≤ 0.001, respectively, ANOVA after normalization to the mean TRPM8 siRNA 0 Gy value of independent experiments, n.t. indicates not significantly different.

Since, on the one hand, cell cycle progression is controlled by Ca^2+^ signaling and on the other hand impaired cell cycle regulation may result in inhibition of DNA repair and radiosensitization [26–29], the effect of TRPM8 knockdown on cell cycle distribution and DNA replication was analyzed in control and irradiated T98G and U251 cells. To this end, the cellular DNA content was measured in permeabilized cells by flow cytometry using the fluorescence dye propidium iodide (Nicoletti protocol, Figure 5A-5C) and the DNA replication by the base analogon 5-ethynyl-2′-deoxyuridine (EdU) incorporation (Figure 5D-5H). In unirradiated cells, TRPM8 knockdown decreased G_1_ population and increased G_2_ population in U251 and decreased S population and increased hyper G population in T98G cells (Figure 5B, 5C, open bars) suggestive of a TRPM8 function in mitosis.

### Irradiation induces TRPM8-dependent S and TRPM8-independent G_2_/M cell cycle arrest, as well as a partially TRPM8-dependent accumulation of cells with increased DNA content

In both glioblastoma lines, irradiation increased the G_2_ and decreased the G_1_ population irrespective of TRPM8 knockdown (compare 1^st^ with 2^nd^ and 3^rd^ with 4^th^ bar in the 1^st^ and 3^rd^ diagram of Figure 5B, 5C). In addition, radiation induced in nt siRNA- but not in TRPM8 siRNA-transfected cells an increase in S (U251 and T98G) population. Together this indicates a radiation-induced S and G_2_/M cell cycle arrest with the former being dependent on TRPM8. Moreover, in T98G but not in U251 cells, radiation and TRPM8 knockdown elevated the hyper G population in an additive manner. In U251 cells, the TRPM8 knockdown increased hyper G only following irradiation (Figure 5B, 5C, 4^th^ diagrams). The hyper G population represents cells with elevated DNA content. An increase in this population, therefore, hints to an impairment of mitosis or cytokinesis.

### Irradiated (4 Gy) glioblastoma cells re-enter cell cycle after a time lag of 2 days

To directly test for a function of TRPM8 for the re-entry in mitosis and cell division upon radiation-induced cell cycle arrest, T98G and U251 cells were irradiated (0 or 4 Gy) and further incubated for 48 h. Thereafter, EdU (see above) was incubated for 8 h before EdU content of the DNA was determined by flow cytometry (Figure 5D). Irradiation (1^st^ and 2^nd^ bars in Figure 5E, 5F) resulted in an increase in EdU-negative S (U251 and T98G) and G_2_ phase (U251) cells and in a decrease of EdU-negative G_1_ cells (U251 and T98G, Figure 5E, 5F, 1^st^-3^rd^ diagrams) while having no effect on the S and G_2_ phase cells that incorporated EdU (Figure 5E, 5F, 4^th^-5^th^ diagrams). The latter observation suggests that G_1_/S transition and S phase progression (i.e., DNA replication) seemed to be restored to levels of unirradiated cells already 48-54 h after irradiation with 4 Gy.

### TRPM8 contributes to S phase progression and mitosis

In both lines TRPM8 knockdown increased the EdU-negative S and G_2_ phase cells in irradiated and unirradiated cells (compare 1^st^ with 3^rd^ and 2^nd^ with 4^th^ bars in Figure 5E, 5F, 1^st^-2^nd^ diagrams). In addition, TRPM8 knockdown decreased in unirradiated U251 and T98G cells as well as in irradiated T98G cells the EdU-negative G_1_ population (compare 1^st^ with 3^rd^ and 2^nd^ with 4^th^ bars in Figure 5E, 5F, 1^st^-2^nd^ diagrams). Moreover, TRPM8 knockdown decreased in U251 cells the EdU-positive S and G_2_ population and in T98G cells the EdU-positive S population irrespective of irradiation (Figure 5E, 5F, 4^th^ and 5^th^ diagrams). The parallel increase and decrease in EdU-negative G_2_ and G_1_ population, respectively, again points to a role of TRPM8 in mitosis. Moreover, the TRPM8 knockdown-associated increase in EdU-negative S cells hints to an additional function of TRPM8 in S phase progression. Computing the ratio between EdU-positive and EdU-negative cells revealed that irradiation alone did not change this ratio in nt siRNA-transfected U251 and T98G cells (compare 1^st^ and 2^nd^ bars in Figure 5G, 5H) again pointing to a restored DNA replication 48-54 h after irradiation. TRPM8 knockdown, in contrast, decreased the ratio between EdU-positive and EdU-negative cells (compare 1^st^ and 3^rd^ bars in Figure 5F, 5H). In U251, but not in T98G cells, combined irradiation and TRPM8 knockdown further decreased this ratio (compare 3^rd^ and 4^th^ bar in Figure 5F, 5H).

Together, the cell cycle data suggest a functional significance of TRPM8 channels in cell cycle control of unirradiated and irradiated glioblastoma cells. In both cell lines, TRPM8 knockdown increased the hyper G population of irradiated cells (see Figure 5B, 5C, 4^th^ diagrams) which points to an impairment of mitosis or cytokinesis.

### TRPM8 signals to the cdc2 subunit of the mitosis-promoting factor

**Figure 6:**
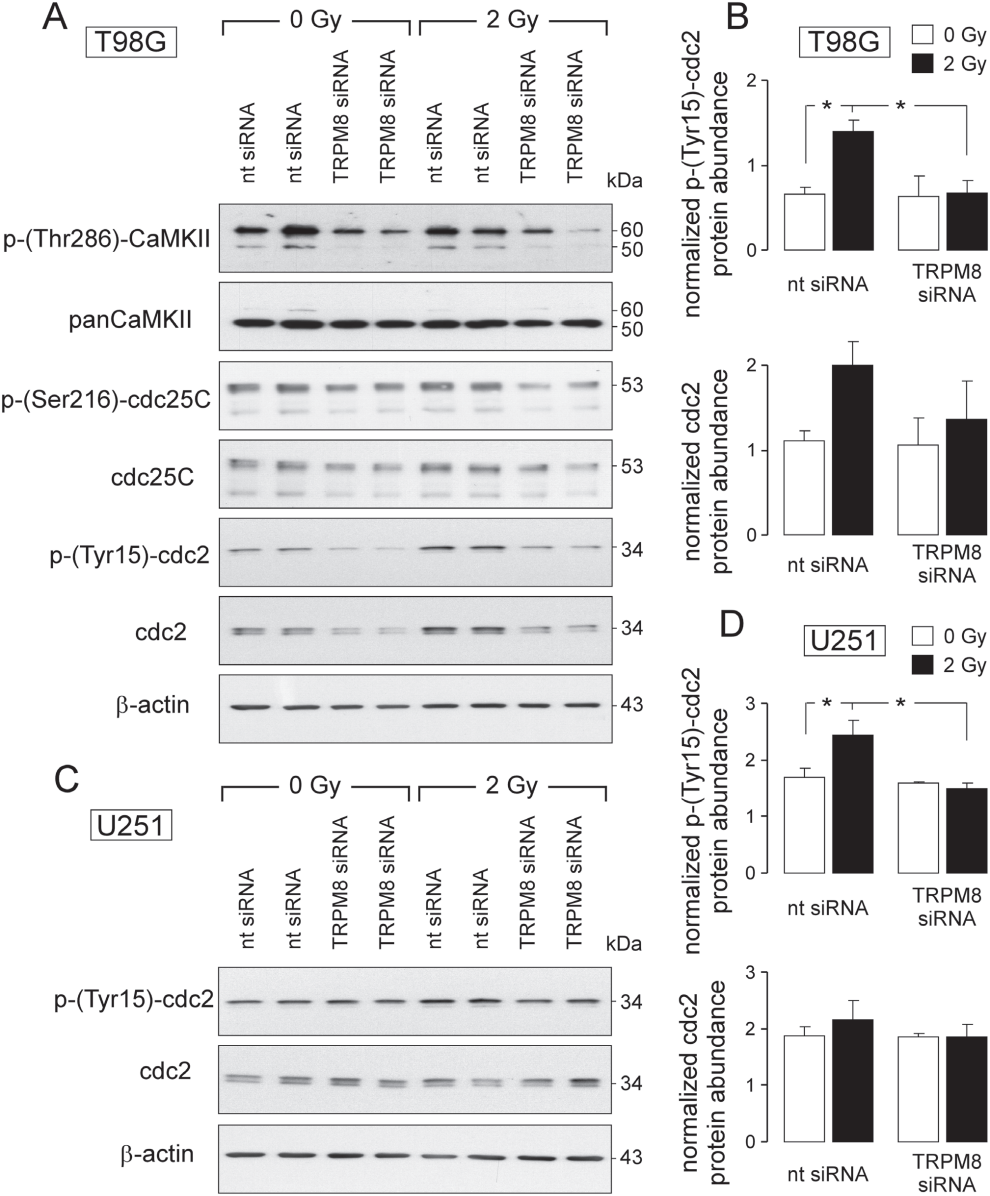
TRPM8-mediated signaling contributes to radiation-induced inhibition of the mitosis-promoting complex **(A, C)** Immunoblots of whole cell lysates from T98G (A) and U251 (C) cells (given are two individual samples for each condition) probed against p-(Thr286)-CaMKII, panCaMKII, p-(Ser216)-cdc25C, cdc25C (T98G), p-(Tyr15)-cdc2, cdc2 and for loading control against β-actin (T98G and U251). Cells were either transfected with nontargeting siRNA (nt-siRNA) or siRNA targeting TRPM8 (TRPM8 siRNA), irradiated with 0 or 2 Gy and lysed 4 h thereafter. **(B, D)** Mean (± SE, n = 4) β-actin-normalized protein abundances of p-(Tyr15)-cdc2 (top) and cdc2 (botton) in nt siRNA or TRPM8 siRNA-transfected T98G (B) and U251 (D) cells 4 h after irradiation with 0 Gy (open bars) or 4 Gy (closed bars) ^*^ indicates p ≤ 0.05, ANOVA.

To identify signaling pathways involved in the regulation of cell cycle by TRPM8, abundance and activity of Ca^2+^/calmodulin-dependent kinases-II isoforms (CaMKII), reported Ca^2+^ downstream effectors and their targets, the phosphatases cdc25C and cdc2 [30], were determined by immunoblotting in nt- or TRPM8 siRNA-transfected T98G cells 4 h after irradiation with 0 or 2 Gy. TRPM8 knockdown decreased CaMKIIs activity in control and irradiated cells as deduced from a decline in the Thr286-phosphorylated kinase proteins (Figure 6A, 1^st^ and 2^nd^ blot). Moreover, TRPM8 knockdown decreased the protein abundance of both, total and inactive, i.e., Ser216-phosporylated cdc25C in control and irradiated T98G cells (Figure 6A, 3^rd^ and 4^th^ blot). In addition, irradiation increased the abundance of the inactive, i.e. Tyr15-phosphorylated cdc2 subunit of the mitosis-promoting factor in nt- but not TRPM8 siRNA-transfected T98G cells (Figure 4A, 5^th^ blot and Figure 6B, top). The effects of radiation and TRPM8 knockdown on abundance of total cdc2 protein were similar in trend (yet without reaching statistical significance, Figure 6A, 6^th^ blot and Figure 6B, bottom). Likewise, radiation (2 Gy) induced within 4 h an increase in abundance of Tyr15-phosphorylated cdc2 in nt- but not in TRPM8 siRNA-transfected U251 cells (Figure 6C, 1^st^ blot and Figure 4D, top). Here, radiation of TRPM8 knockdown did not affect abundance of total cdc2 protein (Figure 6C, 2^nd^ blot and Figure 6D, bottom). The data acquired in T98G cells suggest that TRPM8 is required for the activity of the CaMKII isoforms as well as the inactivating phosphorylation and abundance of the CaMKII target cdc25C. In T98G and U251, TRPM8 knockdown consistently prevented radiation-induced inactivation of cdc2 by phosphorylation at Tyr15. Since p-(Tyr15)-cdc2 is a substrate of the phosphatase cdc25C [30], these data suggest a TRPM8-triggered signaling to cdc2 involving CaMKII and cdc25C in irradiated glioblastoma cells.

In summary, the data demonstrate that glioblastoma cells express TRPM8 channels, which exert pivotal functions in cell cycle control, prevention of apoptotic cell death, migration and chemotaxis, DNA repair and radioresistance.

## DISCUSSION

The present study confirms previous data [10, 11] showing that TRPM8 channels constitute a Ca^2+^ entry pathway in glioblastoma cells and that TRPM8 channel activity stimulates glioblastoma cell migration. The new findings of our study are: i) TRPM8 signaling regulates cell cycle and represses apoptotic cell death. ii) Ionizing radiation with doses used in the clinic for fractionated radiation therapy of glioblastoma patients induces upregulation of TRPM8 function. iii) Elevated TRPM8 function, in turn, confers radioresistance. iv) The latter might result from the observed dependence of cell cycle control during DNA damage response and DNA double strand break repair on TRPM8 signaling.

Combined, our observations suggest that TRPM8 promotes brain invasion and confers radioresistance of glioblastoma cells. This assumption is in contradiction to a retrospective clinical study which concluded that higher TRPM8 mRNA abundance in the tumor associates with better overall survival of glioblastoma patients [2]. The interpretation of these clinical data, however, is largely limited due to low patient number (n = 33) which does not allow multivariate statistical testing (considering established prognostic and predictive parameters). Nevertheless, this previous study [2] demonstrated that among 20 TRP channels subtypes of the melastatin (TRPM), vanilloid (TRPV), and canonical (TRPC) family tested, TRPM8 showed highest upregulation in glioblastoma as compared to normal brain tissue strongly suggesting a pivotal function of TRPM8 in gliomagenesis.

### Radiation-induced Ca^2+^ signaling

In addition to glioblastoma, chronic myeloid and T cell leukemia cells have been reported to respond to ionizing radiation with Ca^2+^ signaling that utilizes Ca^2+^-permeable TRP channels [27, 28]. These TRP channels have been suggested to regulate cell cycle progression and contribute to survival in concert with voltage-gated Kv3.4 [29] or Ca^2+^-activated IK K^+^ channels [23, 26]. By re- or hyperpolarizing the plasma membrane, voltage-gated or Ca^2+^-activated K^+^ channels counteract Ca^2+^ entry-generated membrane depolarization. By doing so, these K^+^ channels maintain locally, i.e., in close vicinity to a Ca^2+^ entry pathway, a high inwardly directed electrochemical Ca^2+^ gradient across the plasma membrane. Hence, K^+^ channels regulate the activity of voltage-gated or voltage-dependent Ca^2+^ entry pathways and are, therefore, important players of the Ca^2+^-signalosome (for review see [1]).

### Interaction between TRPM8, IK and BK K^+^ channels

In glioblastoma, ionizing radiation has been shown to activate BK [22, 24] and IK Ca^2+^-activated K^+^ channels [23]. Activities of both channels signal back to Ca^2+^ or biochemical signaling [26]. For instance, radiation-induced BK K^+^ channel electrosignaling has been demonstrated to be pivotal for the activation of downstream Ca^2+^ effector proteins such as the CaMKII isoforms and glioblastoma cell migration [22]. Radiation-induced IK electrosignaling, on the other hand, is reportedly required for cell cycle control, DNA damage repair and clonogenic survival [23]. Since TRPM8 function - as suggested by the present study - seems to be needed for both, migration and survival of irradiated cells, it is tempting to speculate that IK and BK K^+^ channels closely interact with TRPM8 in glioblastoma cells. The cation permeability of the strongly outwardly rectifying TRPM8 channels decreases with hyperpolarizing voltages (for review see [15]). Therefore, a plausible scenario might be that IK and BK K^+^ channel activities hyperpolarize the plasma membrane and shut down TRPM8 channel-mediated Ca^2+^ influx. Decrease of Ca^2+^ influx, however, deactivates the Ca^2+^-activated BK and IK K^+^ channels resulting in membrane re-depolarization. Depolarization again increases TRPM8-mediated Ca^2+^-entry and reactivation of the K^+^ channels. Thus, reciprocal and interdependent activity of IK, BK, and TRPM8 may generate membrane voltage and Ca^2+^ oscillations specifically recognized by certain Ca^2+^ effector proteins such as CaMKII isoforms (for review see [1]). CaMKIIs, in turn, reportedly regulate glioblastoma cell migration [22, 31, 32] and mitosis [33].

### TRPM8 and cell cycle control

The present study demonstrated an involvement of TRPM8 signaling in inhibition of the cdc2 subunit of the mitosis promoting factor possibly via inhibitory phosphorylation of the cdc25C phosphatase by CaMKII (see Figure 6).

Previous work of our laboratory demonstrated radiation-induced activation of CaMKII and hypermigration of T98G glioblastoma cells which was inhibited by the CaMKII blocker KN93 [22]. Notably, CaMKII activation and hypermigration, both, required radiation-induced BK K^+^ channel activity suggesting BK K^+^ channel signaling upstream of CaMKII [22]. In the present study, TRPM8 activity preceded BK K^+^ channel activation (see Figure 1), radiation stimulated TRPM8 activity (see Supplementary Figure 2), and TRPM8 knockdown prevented basal and radiation-induced CaMKII activity (see Figure 6). Combined, this argues for a TRPM8 - BK K^+^ channel - CaMKII signaling axis.

TRPM8 knockdown prevented radiation-induced inhibition of cdc2 activity as evident from a decrease in abundance of Tyr15-phosphorylated cdc2. This suggests that TRPM8 knockdown leads to an overriding of radiation-induced G_2_/M arrest. Accordingly, TRPM8 knockdown and irradiation induced in flow cytometry experiments an increase in hyper G population indicative of an impairment of mitosis or cytokinesis (see Figure 5B, 5C). It might be speculated that inhibition of G_2_/M cell cycle arrest by TRPM8 knockdown results in premature mitosis, i.e., mitosis with residual DNA damage eventually leading to mitotic catastrophe. The latter probably contributed to the observed radiosensitizing effect of TRPM8 knockdown. The effect of TRPM8 knockdown on radiation-stimulated increase in phospho-(Tyr15)-cdc2 abundance, however, was lower in U251 than in T98G cells (see Figure 6B, 6D) while the radiosensitizing effect was much higher in U251 as compared to T98G cells. At least in U251 cells, this observation suggests further TRPM8-regulated radioprotective mechanisms. Among those might be a function of TRPM8 in DNA damage repair as deduced from the TRPM8 knockdown-associated increase in residual number of radiation-induced γH2AX foci. Notably, this increase was higher in U251 than in T98G cells consistent with the degree of TRPM8 knockdown-associated radiosensitization in both glioblastoma lines.

A function of Ca^2+^-permeable TRP channels for radiation-induced cdc2 inactivation and G_2_/M cell cycle arrest has been demonstrated in leukemia cells [27–29] suggesting that regulation of cdc2 by TRP channel-mediated Ca^2+^ signals is a more common phenomenon and not limited to glioblastoma. In unirradiated T98G and U251 cells, TRPM8 knockdown delayed mitosis as evident from decrease and increase in EdU negative G_1_ and G_2_ population, respectively, and inhibited S progression as apparent from a decrease in EdU incorporation. Combined, these data suggest that TRPM8-signaling regulates S progression and mitosis beyond radiation-induced G_2_/M cell cycle arrest. Notably, inhibition of IK Ca^2+^-activated K^+^ channels in unirradiated and irradiated T98G cells exerted effects on cell cycle progression [23] that differed from those observed upon TRPM8 knockdown pointing to a highly complex regulation of cell cycle by electro- and Ca^2+^ signaling in glioblastoma cells.

### TRPM8 activation in glioblastoma cells

In brain tumors the cold sensor TRPM8 is not exposed to low temperature, which is required to activate the channel in peripheral neurons. In addition, our experimental settings (e.g, migration, colony formation assays) did not subject the cells to any changes in osmolarity, suggesting that the documented osmosensing function of TRPM8 [18] can also not account for the observed TRPM8 activity in glioblastoma cells. TRPM8 channel function is reportedly modulated by G-protein coupled receptors [34], phosphoinosites [15], serine/threonine [35] and tyrosine kinases/phosphatases [36], as well as UBA1-dependent ubiquitination [19]. Moreover, protein-protein interactions with the two-transmembrane domain protein Pirt [37] and the TRP channel-associated factors (TCAFs) [38] reportedly promote trafficking and/or modulate activity of TRPM8. Finally, picomolar concentrations of testosterone have been shown to stimulate openings of the purified TRPM8 channel protein in planar lipid bilayers [16]. Combined, these data suggest a highly complex regulation of TRPM8 surface expression and activity that might shift the set point of temperature-dependent activation towards body temperature. The exact molecular mechanisms leading to activation of TRPM8 in glioblastoma, however, are far from being understood.

### Upregulation of TRPM8 expression by glioblastoma cells

Reportedly, TRPM8 is a target gene of the androgen receptor and p53 [14, 19]. The documented upregulation of the androgen receptor in glioblastoma [39] might therefore contribute to TRPM8 overexpression in the brain tumor. In U251 glioblastoma cells, downregulation of Musashi-1 (MSI1) was associated with upregulation of TRPM8 (see Figure 3A, 3B). This might hint to a possible negative regulation of TRPM8 by the RNA binding protein musashi-1. Querying the Cancer-Genome-Atlas for MSI1 and TRPM8 mRNA abundances, however, did not confirm such an association in glioblastoma patient specimens (data not shown) suggesting that this phenomenon might be restricted to U251 cells. In the present study, TRPM8 mRNA abundance increased significantly in T98G and U251 glioblastoma cells and not quite significantly in U-87MG cells during fractionated radiation (see Supplementary Figure 2C-2F) Similarly, fractionated radiation increased TRPM8 immunofluorescence in an orthotopic U-87MG-Katushka glioblastoma mouse model *in vivo* (see Supplementary Figure 2G). This suggests either selection of TRPM8 overexpressing and resistant glioblastoma cell clones or an adaptive response. The latter might in theory result from stabilization of p53. T98G and U251, which are p53-mutated [40], however, show higher fractionated radiation-induced increase in TRPM8 than p53 wildtype U-87MG cells, an observation which does not support a p53-mediated TRPM8 upregulation.

## CONCLUSIONS

The present *in vitro* study proposes a possible functional significance of Ca^2+^-permeable TRPM8 nonselective cation channels for glioblastoma survival, local tumor invasion, and radioresistance. Therefore, targeting TRPM8 alone or in combination with radiotherapy, possibly might be a promising new strategy for future anti-glioblastoma therapy. New and more specific pharmacological TRPM8 inhibitors are available [41, 42], antibodies binding to extracellular TRPM8 protein and inhibiting TRPM8 function have been developed [43] suggesting that TRPM8-targeting therapy, at least in theory, might be feasible. Whether or not TRPM8-targeting alone or in combination with radiotherapy has tolerable side effects and does effectively prolong survival has to await future preclinical studies in orthotopic glioblastoma animal models.

## MATERIALS AND METHODS

### Cell culture and transfection

Human T98G, U-87MG (both form ATTC) and U251 (kindly provided by Dr. Luiz Penalva, San Antonio, TX) glioblastoma cells were grown in 10% fetal calf serum (FCS)-supplemented RPMI-1640 (T98G and U-87MG) or DMEM (4500 mg glucose/l, U251) medium. Exponentially growing U251 and T98G cells were reversely transfected with stealth siRNAs (Thermo Fischer Scientific, Waltham, MA, U.S.A) specific for TRPM8 (HSS128188, HSS128189, HSS128190) and MSI1 (HSS106732, HSS106733, HSS106734), or with nt siRNA (*Silencer*® Select Negative Control No. 1 siRNA, #4390844, Ambion™, Thermo Fischer Scientific). Detached T98G and U251 cells (250,000 in 2.5 ml RPMI-1640/10% FCS and DMEM (4500 mg glucose/l/10% FCS medium), respectively, were added to 500 μl of pre-incubated (20 min/RT) Opti-MEM medium containing RNAiMAX lipofectamine (6 μl, Invitrogen Life Technologies, Carlsbad, CA, USA) and siRNA (25 nM final concentration).

### Quantitative RT-PCR

Messenger RNAs of TRPM8 siRNA-, MSI1 siRNA- or nt siRNA-transfected U251 and T98G cells (48-72 h after transfection) were isolated (Qiagen RNA extraction kit, Hilden, Germany) and reversely transcribed in cDNA (Transcriptor First Strand cDNA Synthesis Kit, Roche, Mannheim, Germany). TRPM8-, MSI1 (Musashi-1)-, as well as housekeeper β-actin (ACTB)-, pyruvate dehydrogenase beta (PDHB)-, and glyceraldehyde-3-phosphate dehydrogenase (GAPDH)-specific fragments were amplified by the use of SYBR Green-based quantitative real-time PCR (QuantiTect Primer Assays QT00038906, QT00003780, QT00095431, QT00031227, and QT01192646 and QuantiFast SYBR Green PCR Kit, Qiagen) in a Roche LightCycler Instrument (Roche, Mannheim, Germany). mRNA abundances were normalized to the geometrical mean abundance of the three housekeepers.

### Patch clamp recording

On-cell currents of semi-confluent T98G cells were elicited by 41 voltage square pulses (700 ms each) delivered in 5 mV increments from 0 mV holding potential to voltages between -100 mV and +100 mV. Voltages refer to the intracellular face of the plasma membrane. Flow of positive charge out of the cells (or the counter flow of anions) is defined as positive current and depicted as upward deflection of the current tracings. Cells were superfused at 37°C with Ca^2+^-containing NaCl solution (in mM: 125 NaCl, 32 HEPES, 5 KCl, 5 D-glucose, 1 MgCl_2_, 1 CaCl_2_, titrated with NaOH to pH 7.4). This NaCl solution was also used for the pipette solution further containing 0 or 10 μM icilin (in 0.1% DMSO). Macroscopic on-cell currents were analyzed by averaging the currents between 100 and 700 ms of each square pulse.

### Migration assay

For transfilter chemotaxis, the lower and upper chamber of a CIM-Plate 16 (Roche, Mannheim, Germany) were filled with 160 μl (lower chamber) and 100 μl (upper chamber) of RPMI-1640 medium containing 5% and 1% FCS, respectively, equilibrated at 37°C and 5% CO_2_ for 30-60 min. After CO_2_ equilibration and resetting the impedance to zero, 100 μl of cell suspension containing 40.000 TRPM8- or nt siRNA-treated T98G cells (48 h after transfection) in RPMI-1640/1% FCS were added to the upper chamber. After sedimentation and adherence of the cells, migration was analyzed. The upper and lower chamber additionally contained icilin, or the TRPM8 inhibitor N-(4-tert-butylphenyl)-4-(3-chloropyridin-2-yl)piperazine-1-carboxamide, BCTC (all 0 or 10 μM in 0.1% DMSO). Transfilter migration was assessed by measuring the electrical impedance increase between electrodes that cover the lower surface of the filter membrane (i.e., the bottom of the upper chamber) and the reference electrode at the bottom of the lower chamber. Upon transfilter migration, cells adhere to the electrode at the lower surface of the filter membrane and, thereby, increase the impedance.

For cell migration during wound healing, control (T98G, U-87MG) and TRPM8- or nt siRNA-treated T98G glioblastoma cells were plated in 35 mm dishes (120.000 cells per insert) with culture inserts (Ibidi, Martinsried, Germany). After monolayer formation, cells were pretreated (30 min) with TRPM8 activator icilin or inhibitor BCTC (all 0 or 10 μM in 0.1% DMSO) in phenol red-free 20 mM HEPES (pH 7.4)-supplemented RPMI-1640 medium. Migration assay was started by removing the inserts to create a wound of approximately 500 μm in width. Thereafter (0 - 18 h), 3 fields of the wound were photographed with an inverse light microscope every 30 min (Axio Observer.Z1, 10x, Zeiss, Jena). For each field 5 cells were tracked (Manual Tracking, ImageJ) and the velocity was calculated.

### Fura-2 fluorescence imaging of _free_[Ca^2+^]_i_

Fluorescence measurements were performed at 37°C using an inverted phase-contrast microscope (Axiovert 100; Zeiss, Oberkochen, Germany). Fluorescence was evoked by a filter wheel (Visitron Systems, Puchheim, Germany)-mediated alternative excitation at 340/26 or 387/11 nm (AHF, Analysentechnik, Tübingen, Germany). Excitation and emission light was deflected by a dichroic mirror (409/LP nm beam splitter, AHF) into the objective (Fluar x40/1.30 oil; Zeiss) and transmitted to the camera (Visitron Systems), respectively. Emitted fluorescence intensity was recorded at 587/35 nm (AHF). Excitation was controlled and data acquired by Metafluor computer software (Universal Imaging, Downingtown, PA, USA). The 340/380-nm fluorescence ratio was used as a measure of cytosolic free Ca^2+^ concentration (_free_[Ca^2+^]_i_). TRPM8- , MSI1- or nt siRNA-transfected U251 cells (24 h (MSI1) and 48 h (TRPM8) after transfection) were incubated with fura-2/AM (2 μM for 30 min at 37°C; Molecular Probes, Goettingen, Germany) in DMEM/10% FCS medium, respectively. Steady state _free_[Ca^2+^]_i_ was recorded in Ca^2+^-containing NaCl solution (see above) before and during superfusion of icilin (10 μM).

### Ionizing radiation

Exponentially growing T98G, and U251 cells were irradiated (single dose of 0, 2, 4 and 6 Gy) with 6 MV photons by using a linear accelerator (LINAC SL25 Philips) at a dose rate of 4 Gy/min at room temperature. Open-field irradiation was applied. For a sufficient dose buildup in the plane of the cells, a RW3 slab (1 cm thick) was placed above the cells. Energy deposition at the cell plane was calibrated by film dosimetry. Following IR, cells were post-incubated in the respective cell culture media for 4 h (immunoblotting), 24 h (immunofluorescence microscopy), 48 h (flow cytometry, Nicoletti protocol), 54 h (flow cytometry, EdU incorporation), or 2-3 weeks (colony formation assay).

### Colony formation assay

To test for clonogenic survival, TRPM8-, or nt siRNA-transfected U251 and T98G cells (48 h after transfection) were irradiated (0, 2, 4 or 6 Gy) in the respective cell culture media. Twenty-four hours after IR, 400 and 600 (T98G) or 1200 (U251) cells were re-seeded on 6-well plates and grown for further 2-3 weeks. Clusters of ≥ 50 cells were defined as colony. Colonies were counted manually. The plating efficiency was defined by dividing the number of colonies by the number of plated cells. Survival fractions as calculated by dividing the plating efficiency of the irradiated cells by those of the unirradiated controls were fitted by SigmaPlot (Systat Software GmbH, Erkrath, Germany) to the linear quadratic equation S = e^–(α·D + ß·D^2^) with S being the survival fraction, D the radiation dose, and α,·ß tissue constants.

### Immunoblotting

Whole protein lysates were prepared from semiconfluent irradiated (0 or 2 Gy) nt- or TRPM8 siRNA-transfected T98G and U251 cells (48-72 h and 4 h after transfection and irradiation, respectively) using a buffer containing (in mM) 50 HEPES pH 7.5, 150 NaCl, 1 EDTA, 10 sodium pyrophosphate, 10 NaF, 2 Na_3_VO_4_, 1 phenylmethylsulfonylfluorid (PMSF) additionally containing 1% Triton X-100, 5 μg/ml aprotinin, 5 μg/ml leupeptin, and 3 μg/ml pepstatin (all Sigma-Aldrich), and separated by SDS-PAGE under reducing conditions. Segregated proteins were electro-transferred onto PVDF membranes (Roth, Karlsruhe, Germany). Blots were blocked in tris(hydroxymethyl)aminomethane-buffered saline (TBS) buffer containing 0.05% Tween 20 and 5% non-fat dry milk for 1 h at room temperature. The membranes were incubated overnight at 4°C with the following primary antibodies: rabbit anti TRPM8- (Abcam #3243, Biomol Hamburg, Germany, 1:1000), rabbit anti-phospho-CaMKII (Thr286)- (#3361, Cell Signaling, New England Biolabs, Frankfurt, Germany, 1:500), rabbit anti-CaMKII (pan)- (#3362, Cell Signaling, 1:500), rabbit anti-phospho-cdc25C (Ser216)- (#4901, Cell Signaling, 1:1000), rabbit anti-cdc25C (5H9)- (#4688, Cell Signaling, 1:1000), rabbit anti-phospho-cdc2 (Tyr15)- (#9111, Cell Signaling, 1:1000), rabbit anti-cdc2- (#9112, Cell Signaling, 1:1000), or mouse anti- β-actin antibody (#A5441, Sigma Aldrich, Deisenhofen, Germany, 1:30,000). Antibody binding was detected with a horseradish peroxidase (HRP)-linked goat anti-rabbit or HRP-linked goat anti-mouse IgG antibody (Cell Signaling # 7074 and # #7076, respectively; 1:1000 - 1:2000 dilution in TBS-Tween / 5% milk) incubated for 1 h at room temperature and enhanced chemoluminescence (ECL Western blotting analysis system, GE Healthcare/Amersham-Biosciences, Freiburg, Germany). Indicated protein levels were quantified by densitometry using ImageJ software (ImageJ 1.40g NIH, USA).

### Immunofluorescence microscopy of residual DNA double strand breaks

Nt- and TRPM8 siRNA-transfected U251 or T98G cells (1500 cells per well) were seeded on a Millicell EZ SLIDE 8-well glass (Merck Millipore, Tullagreen, Ireland) and grown for 48 h, irradiated with 0 and 4 Gy, and further incubated for 24 h. Thereafter, cells were fixed with 3.7% formaldehyde for 15 min at 21°C followed by three washing steps with phosphate buffered saline (PBS). Next, cells were permeabilized for 10 min at 21°C with 0.1% Triton-X 100 in PBS followed again by three washing steps with PBS. Cells were probed against γH2AX using anti-γH2AX (Ser139) antibody (monoclonal mouse, clone JBW301, #05-636, Merck Millipore, Darmstadt, Germany) in a 1:1000 dilution. Antibody binding was visualized with the Tyramide Signal Amplification Kit (Molecular Probes, Eugene, Oregon, USA). Prior to mounting, nuclei were counterstained with DAPI (Sigma-Aldrich).

### Flow cytometry

To determine ΔΨ_m_ and caspase activity after irradiation T98G and U251 cells were transfected with TRPM8- or nt siRNA, irradiated (48 h after transfection, 0 or 4 Gy) and incubated for further 24 h in the respective cell culture media. To determine ΔΨ_m_, cells were trypsinated, washed and incubated for 30 min at room temperature in Ca^2+^-containing NaCl solution solution (see above) containing the ΔΨ_m_-specific dye tetramethylrhodamine ethyl ester perchlorate (TMRE, 25 nM for 30 min, Invitrogen, Karlsruhe, Germany). To assess caspase activity, cells were detached and incubated with CaspACE ™ FITC-VAD-FMK (5 μM for 30 min in cell culture medium, Promega, Mannheim, Germany). TMRE and CaspaACE-specific fluorescence was measured by flow cytometry (FACS Calibur, Becton Dickinson, Heidelberg, Germany, 488 nm excitation wavelength) in fluorescence channel FL-2 (585/42 nm) and FL-1 (530/30 nm) emission wavelength, respectively.

### Cell cycle analysis

For cell cycle analysis, irradiated TRPM8- or nt siRNA transfected T98G and U251 cells (0 or 4 Gy, 48 h after IR and 96 h after transfection) were permeabilized and stained (30 min at room temperature) with propidium iodide (PI) according to the Nicoletti protocol (containing 0.1% Na-citrate, 0.1% triton X-100, 10 μg/ml PI in RNase-containing phosphate-buffered saline, PBS), and the DNA amount was analyzed by flow cytometry in fluorescence channel FL-3 (>670 nm, linear scale). Data were analyzed with the FCS Express 3 software (De Novo Software, Los Angeles, CA, USA). For EdU incorporation, irradiated TRPM8- or nt-siRNA transfected T98G and U251 cells (0 or 4 Gy, 48 h after IR and 96 h after transfection) were incubated for further 6 h in the respective cell culture media additionally containing the base analogon 5-ethynyl-2’-deoxyuridine (EdU, 5 μM). EdU incorporation was analyzed by the use of an EdU flow cytometry kit (BCK-FC488, baseklick, Tutzing, Germany) after fixing the cells and co-staining the DNA with propidium iodide (PI, Sigma-Aldrich) according the manufacturer's instructions. EdU- and PI-specific fluorescence were recorded by flow cytometry in fluorescence channel FL-1 (log scale) and FL-3 (linear scale), respectively.

## SUPPLEMENTARY MATERIALS FIGURES



## References

[1] Huber SM (2013). Oncochannels. Cell Calcium.

[2] Alptekin M, Eroglu S, Tutar E, Sencan S, Geyik MA, Ulasli M, Demiryurek AT, Camci C (2015). Gene expressions of TRP channels in glioblastoma multiforme and relation with survival. Tumour Biol.

[3] Tsavaler L, Shapero MH, Morkowski S, Laus R (2001). Trp-p8, a novel prostate-specific gene, is up-regulated in prostate cancer and other malignancies and shares high homology with transient receptor potential calcium channel proteins. Cancer Res.

[4] Liu J, Chen Y, Shuai S, Ding D, Li R, Luo R (2014). TRPM8 promotes aggressiveness of breast cancer cells by regulating EMT via activating AKT/GSK-3beta pathway. Tumour Biol.

[5] Yee NS, Li Q, Kazi AA, Yang Z, Berg A, Yee RK (2014). Aberrantly over-expressed TRPM8 channels in pancreatic adenocarcinoma: correlation with tumor size/stage and requirement for cancer cells invasion. Cells.

[6] Yu S, Xu Z, Zou C, Wu D, Wang Y, Yao X, Ng CF, Chan FL (2014). Ion channel TRPM8 promotes hypoxic growth of prostate cancer cells via an O2-independent and RACK1-mediated mechanism of HIF-1alpha stabilization. J Pathol.

[7] Wang Y, Yang Z, Meng Z, Cao H, Zhu G, Liu T, Wang X (2013). Knockdown of TRPM8 suppresses cancer malignancy and enhances epirubicin-induced apoptosis in human osteosarcoma cells. Int J Biol Sci.

[8] Xiao N, Jiang LM, Ge B, Zhang TY, Zhao XK, Zhou X (2014). Over-expression of TRPM8 is associated with poor prognosis in urothelial carcinoma of bladder. Tumour Biol.

[9] Borrelli F, Pagano E, Romano B, Panzera S, Maiello F, Coppola D, De Petrocellis L, Buono L, Orlando P, Izzo AA (2014). Colon carcinogenesis is inhibited by the TRPM8 antagonist cannabigerol, a Cannabis-derived non-psychotropic cannabinoid. Carcinogenesis.

[10] Wondergem R, Ecay TW, Mahieu F, Owsianik G, Nilius B (2008). HGF/SF and menthol increase human glioblastoma cell calcium and migration. Biochem Biophys Res Commun.

[11] Wondergem R, Bartley JW (2009). Menthol increases human glioblastoma intracellular Ca2+, BK channel activity and cell migration. J Biomed Sci.

[12] Yamamura H, Ugawa S, Ueda T, Morita A, Shimada S (2008). TRPM8 activation suppresses cellular viability in human melanoma. Am J Physiol Cell Physiol.

[13] Bidaux G, Flourakis M, Thebault S, Zholos A, Beck B, Gkika D, Roudbaraki M, Bonnal JL, Mauroy B, Shuba Y, Skryma R, Prevarskaya N (2007). Prostate cell differentiation status determines transient receptor potential melastatin member 8 channel subcellular localization and function. J Clin Invest.

[14] Bidaux G, Roudbaraki M, Merle C, Crepin A, Delcourt P, Slomianny C, Thebault S, Bonnal JL, Benahmed M, Cabon F, Mauroy B, Prevarskaya N (2005). Evidence for specific TRPM8 expression in human prostate secretory epithelial cells: functional androgen receptor requirement. Endocr Relat Cancer.

[15] Yudin Y, Rohacs T (2012). Regulation of TRPM8 channel activity. Mol Cell Endocrinol.

[16] Asuthkar S, Demirkhanyan L, Sun X, Elustondo PA, Krishnan V, Baskaran P, Velpula KK, Thyagarajan B, Pavlov EV, Zakharian E (2015). The TRPM8 protein is a testosterone receptor: II. Functional evidence for an ionotropic effect of testosterone on TRPM8. J Biol Chem.

[17] Asuthkar S, Elustondo PA, Demirkhanyan L, Sun X, Baskaran P, Velpula KK, Thyagarajan B, Pavlov EV, Zakharian E (2015). The TRPM8 protein is a testosterone receptor: I. Biochemical evidence for direct TRPM8-testosterone interactions. J Biol Chem.

[18] Quallo T, Vastani N, Horridge E, Gentry C, Parra A, Moss S, Viana F, Belmonte C, Andersson DA, Bevan S (2015). TRPM8 is a neuronal osmosensor that regulates eye blinking in mice. Nat Commun.

[19] Asuthkar S, Velpula KK, Elustondo PA, Demirkhanyan L, Zakharian E (2015). TRPM8 channel as a novel molecular target in androgen-regulated prostate cancer cells. Oncotarget.

[20] Chodon D, Guilbert A, Dhennin-Duthille I, Gautier M, Telliez MS, Sevestre H, Ouadid-Ahidouch H (2010). Estrogen regulation of TRPM8 expression in breast cancer cells. BMC Cancer.

[21] Huber SM, Butz L, Stegen B, Klumpp L, Klumpp D, Eckert F (2015). Role of ion channels in ionizing radiation-induced cell death. Biochim Biophys Acta.

[22] Steinle M, Palme D, Misovic M, Rudner J, Dittmann K, Lukowski R, Ruth P, Huber SM (2011). Ionizing radiation induces migration of glioblastoma cells by activating BK K+ channels. Radiother Oncol.

[23] Stegen B, Butz L, Klumpp L, Zips D, Dittmann K, Ruth P, Huber SM (2015). Ca2+-activated IK K+ channel blockade radiosensitizes glioblastoma cells. Mol Cancer Res.

[24] Edalat L, Stegen B, Klumpp L, Haehl E, Schilbach K, Lukowski R, Kuhnle M, Bernhardt G, Buschauer A, Zips D, Ruth P, Huber SM. BK (2016). K+ channel blockade inhibits radiation-induced migration/brain infiltration of glioblastoma cells. Oncotarget.

[25] Rubner Y, Muth C, Strnad A, Derer A, Sieber R, Buslei R, Frey B, Fietkau R, Gaipl US (2014). Fractionated radiotherapy is the main stimulus for the induction of cell death and of Hsp70 release of p53 mutated glioblastoma cell lines. Radiat Oncol.

[26] Stegen B, Klumpp L, Misovic M, Edalat L, Eckert M, Klumpp D, Ruth P, Huber SM (2016). K+ channel signaling in irradiated tumor cells. Eur Biophys J.

[27] Heise N, Palme D, Misovic M, Koka S, Rudner J, Lang F, Salih HR, Huber SM, Henke G (2010). Non-selective cation channel-mediated Ca2+-entry and activation of Ca2+/calmodulin-dependent kinase II contribute to G2/M cell cycle arrest and survival of irradiated leukemia cells. Cell Physiol Biochem.

[28] Klumpp D, Misovic M, Szteyn K, Shumilina E, Rudner J, Huber SM (2016). Targeting TRPM2 channels impairs radiation-induced cell cycle arrest and fosters cell death of T cell leukemia cells in a Bcl-2-dependent manner. Oxid Med Cell Longev.

[29] Palme D, Misovic M, Schmid E, Klumpp D, Salih HR, Rudner J, Huber SM (2013). Kv3.4 potassium channel-mediated electrosignaling controls cell cycle and survival of irradiated leukemia cells. Pflugers Arch.

[30] Hutchins JR, Dikovskaya D, Clarke PR (2003). Regulation of Cdc2/cyclin B activation in Xenopus egg extracts via inhibitory phosphorylation of Cdc25C phosphatase by Ca2+/calmodulin-dependent protein kinase II. Mol Biol Cell.

[31] Cuddapah VA, Sontheimer H (2010). Molecular interaction and functional regulation of ClC-3 by Ca2+/calmodulin-dependent protein kinase II (CaMKII) in human malignant glioma. J Biol Chem.

[32] Cuddapah VA, Turner KL, Seifert S, Sontheimer H (2013). Bradykinin-induced chemotaxis of human gliomas requires the activation of KCa3.1 and ClC-3. J Neurosci.

[33] Cuddapah VA, Habela CW, Watkins S, Moore LS, Barclay TT, Sontheimer H (2012). Kinase activation of ClC-3 accelerates cytoplasmic condensation during mitotic cell rounding. Am J Physiol Cell Physiol.

[34] Bavencoffe A, Gkika D, Kondratskyi A, Beck B, Borowiec AS, Bidaux G, Busserolles J, Eschalier A, Shuba Y, Skryma R, Prevarskaya N (2010). The transient receptor potential channel TRPM8 is inhibited via the alpha 2A adrenoreceptor signaling pathway. J Biol Chem.

[35] Mandadi S, Armati PJ, Roufogalis BD (2011). Protein kinase C modulation of thermo-sensitive transient receptor potential channels: implications for pain signaling. J Nat Sci Biol Med.

[36] Morgan K, Sadofsky LR, Crow C, Morice AH (2014). Human TRPM8 and TRPA1 pain channels, including a gene variant with increased sensitivity to agonists (TRPA1 R797T), exhibit differential regulation by SRC-tyrosine kinase inhibitor. Biosci Rep.

[37] Tang M, Wu GY, Dong XZ, Tang ZX (2016). Phosphoinositide interacting regulator of TRP (Pirt) enhances TRPM8 channel activity *in vitro* via increasing channel conductance. Acta Pharmacol Sin.

[38] Gkika D, Lemonnier L, Shapovalov G, Gordienko D, Poux C, Bernardini M, Bokhobza A, Bidaux G, Degerny C, Verreman K, Guarmit B, Benahmed M, de Launoit Y (2015). TRP channel-associated factors are a novel protein family that regulates TRPM8 trafficking and activity. J Cell Biol.

[39] Yu X, Jiang Y, Wei W, Cong P, Ding Y, Xiang L, Wu K (2015). Androgen receptor signaling regulates growth of glioblastoma multiforme in men. Tumour Biol.

[40] Gomez-Manzano C, Fueyo J, Kyritsis AP, McDonnell TJ, Steck PA, Levin VA, Yung WK (1997). Characterization of p53 and p21 functional interactions in glioma cells en route to apoptosis. J Natl Cancer Inst.

[41] Ohmi M, Shishido Y, Inoue T, Ando K, Fujiuchi A, Yamada A, Watanabe S, Kawamura K (2014). Identification of a novel 2-pyridyl-benzensulfonamide derivative, RQ-00203078, as a selective and orally active TRPM8 antagonist. Bioorg Med Chem Lett.

[42] Lehto SG, Weyer AD, Zhang M, Youngblood BD, Wang J, Wang W, Kerstein PC, Davis C, Wild KD, Stucky CL, Gavva NR (2015). AMG2850, a potent and selective TRPM8 antagonist, is not effective in rat models of inflammatory mechanical hypersensitivity and neuropathic tactile allodynia. Naunyn Schmiedebergs Arch Pharmacol.

[43] Miller S, Rao S, Wang W, Liu H, Wang J, Gavva NR (2014). Antibodies to the extracellular pore loop of TRPM8 act as antagonists of channel activation. PLoS One.

