# Supplementary material for: TRPM8 is required for survival and radioresistance of glioblastoma cells
Source: Oncotarget. 2017 Sep 30;8(56):95896–913. doi: 10.18632/oncotarget.21436 (PMC5707069; doi:10.18632/oncotarget.21436)
Supplement: Supplementary file 1 [file oncotarget-08-95896-s001.pdf]

TRPM8 is required for survival and radioresistance of glioblastoma cells

SUPPLEMENTARY MATERIALS

Glioblastoma express TRPM8

The present study queried the glioblastoma multiforme database of The-Cancer-Genome-Atlas (TCGA) to assess the mRNA abundance of TRP channels belonging to the melastatin (TRPM), vanilloid (TRPV), and canonical (TRPC) family. As shown in Supplementary Figure 1A, human glioblastoma specimens were highly abundant in TRPM2, TRPM4, TRPM7, TRPM8, TRPV1, TRPV2, and TRPC1 mRNA. Notably, TRPM8 mRNA abundance could be detected in all specimens but varied considerably between individuals. A similar high variance in TRPM8 expression was apparent on protein level in primary cultures of human glioblastoma specimens (Supplementary Figure 1B). Next we determined the mRNA abundances of TRPM1, TRPM8, TRPV1, TRPV2 and TRRPC6 in different human glioblastoma cell lines by qRT-PCR. Supplementary Figure 1C shows the TRP channel-specific RT-PCR products for the human glioblastoma WK1cells indicating high abundances of TRPV1, TRPV2 and TRPM8 mRNA. Supplementary Figure 1D compares the TRPM8 mRNA abundances between the human glioblastoma cell lines WK1, T98G, U373, A172, U-87MG, DBTRG, and U251.

Methods

Via the cBIOportal Web resource [1, 2], the provisional Glioblastoma-Multiforme TCGA database (http://cancergenome.nih.gov/) was queried for mRNA abundance (Illumina HiSeq 2000 RNA Sequencing platform) of transient receptor potential (TRP) cation channels in the tumor specimens (downloaded 2017-05-05). Primary spheroid and differentiated cultures were obtained from resected glioblastoma tissue (patients gave informed consent and this study has been approved by the local ethic committee under project# 579/2015BO2) which has been dissociated enzymatically and mechanically and plated in Complete NeuroCult™ NS-A Proliferation medium containing 20 ng/ml rhEGF, 10 ng/ml rhbFGF and 0.0002% heparin (STEMCELL Technologies Germany GmbH, Cologne, Germany) and 10% fetal calf serum (FCS)-containing RPMI-1640 medium, respectively. Human WK1 (kindly provided by Dr. Michael Fay, Brisbane, Australia), U373, U251 (kindly provided by Dr. Luiz Penalva, San Antonio, TX), T98G, A172, U-87MG, (ATCC) and DBTRG (Sigma-Aldrich) glioblastoma cell lines were grown in 10% FCS-supplemented RPMI-1640 or DMEM (4500 mg glucose/l, U251) medium. For qRT-PCR, TRPM8-, TRPV1-, TRPV2-, TRPC6-, and housekeeper housekeeper β-actin (ACTB)-, pyruvate dehydrogenase beta (PDHB)-, and glyceraldehyde-3-phosphate dehydrogenase (GAPDH)-specific fragments were amplified by the use QuantiTect Primer Assays QT00038906, QT00046109, QT00035987, QT00037660, QT00095431, QT00031227, and QT01192646, respectively, and normalized to the geometrical mean of the housekeeper mRNAs.

Ionizing radiation up-regulates TRPM8 activity and abundance

In order to analyze the mechanism of TRPM8-mediated radioresistance in glioblastoma, we determined radiation-induced changes in TRPM8 activity and expression (Supplementary Figure 2). A single dose (2 Gy) of ionizing radiation evoked within 2 h an increase in menthol-stimulated Ca^{2+} influx into T98G cells as recorded by fura-2 imaging applying a Ca^{2+}-depletion/repletion protocol (Supplementary Figure 2A, 2B). Furthermore, fractionated irradiation (5 x 2 Gy, i.e. daily fraction of 2 Gy on 5 consecutive days) induced a significant increase in TRPM8 mRNA abundance in T98G and U251 cells (Supplementary Figure 2C-2E). In U-87MG which expressed highest basal TRPM8 mRNA abundance among the tested human glioblatoma cell lines (see Supplementary Figure 1D), the fractionated irradiation induced an increase in TRPM8 mRNA abundance, which was not quite significant (Supplementary Figure 2F). Similar to these *in vitro* experiments, fractionated irradiation (5 × 2 Gy) resulted in an upregulation of TRPM8 protein in orthotopic U-87MG-Katushka *xeno*grafts *in vivo* (Supplementary Figure 2G). Together, the data demonstrate a (fractionated) radiation-induced upregulation of TRPM8 function in human glioblastoma.

Methods

In irradiated (0 or 2 Gy, 1.5-3 h after IR) T98G cells, fura-2 fluorescence ratios were measured during superfusion with EGTA-buffered Ca^{2+}-free NaCl (in mM: 125 NaCl, 32 HEPES, 5 KCl, 5 d-glucose, 1 $MgCl_2$, 0.5

EGTA, titrated with NaOH to pH 7.4) solution and upon wash-in of Ca^{2+} in Ca^{2+}-containing NaCl solution (see main text) additionally containing the TRPM8 agonist menthol (100 µM). Abundances of TRPM8 mRNA in fractionated irradiated (5 x 0 Gy or 5 x 2 Gy) T98G, U251 and U-87MG cells (24 h after the last IR fraction) were analyzed by qRT-PCR as described above and normalized to the GAPDH mRNA abundance. Animal experiments were carried out according to the German animal protection law and approved by the local authorities. Human U-87MG-Katushka cells (kindly provided by Prof. Armin Buschauer, Regensburg, Germany) were orthotopically transplanted into the right striatum of mouse brain (day 1), orthotopic glioblastomas were fractionated irradiated (5 x 0 Gy or 5 x 2 Gy on days 8-12), and mice were sacrificed (day 21), excised brains fixed (2% paraformaldehyd in PBS for 24 h), cryo-protected (30% sucrose in PBS for 24 h), frozen at -80° C in Richard-Allan Scientific™ Neg-50™ Frozen Section Medium (Thermo Scientific, Germany), and cryosectioned (20 µm) essentially as described [3–5]. For TRPM8 protein immunostaining, sections were post-fixed 15 min (4% paraformaldehyde in PBS) and processed as described in the main text using rabbit anti-TRPM8 antibody (Abcam #3243, 1:100 for 2 h at 21°C) or rabbit IgG isotype control antibody (#12-370, Merck/Millipore, 1:100) at the same concentration combined with a FITC-linked goat anti-rabbit IgG detection antibody (NB730-F, Novus Biologicals, R&D Systems, Abingdon, U.K., 1:500 for 2 h at 21°C).

REFERENCES

1. Cerami E, Gao J, Dogrusoz U, Gross BE, Sumer SO, Aksoy BA, Jacobsen A, Byrne CJ, Heuer ML, Larsson E, Antipin Y, Reva B, Goldberg AP, et al. The cBio cancer genomics portal: an open platform for exploring multidimensional cancer genomics data. Cancer Discov. 2012; 2:401-4.
2. Gao J, Aksoy BA, Dogrusoz U, Dresdner G, Gross B, Sumer SO, Sun Y, Jacobsen A, Sinha R, Larsson E, Cerami E, Sander C, Schultz N. Integrative analysis of complex cancer genomics and clinical profiles using the cBioPortal. Sci Signal. 2013; 6:pl1.
3. Steinle M, Palme D, Misovic M, Rudner J, Dittmann K, Lukowski R, Ruth P, Huber SM. Ionizing radiation induces migration of glioblastoma cells by activating BK K^+ channels. Radiother Oncol. 2011; 101:122-6.
4. Stegen B, Butz L, Klumpp L, Zips D, Dittmann K, Ruth P, Huber SM. Ca2+-Activated IK K^+ Channel. Mol Cancer Res. 2015; 13:1283-95.
5. Edalat L, Stegen B, Klumpp L, Haehl E, Schilbach K, Lukowski R, Kuhnle M, Bernhardt G, Buschauer A, Zips D, Ruth P, Huber SM. BK K^+ channel blockade inhibits radiation-induced migration/brain infiltration of glioblastoma cells. Oncotarget. 2016; 7:14259-78. https://doi.org/10.18632/oncotarget.7423.

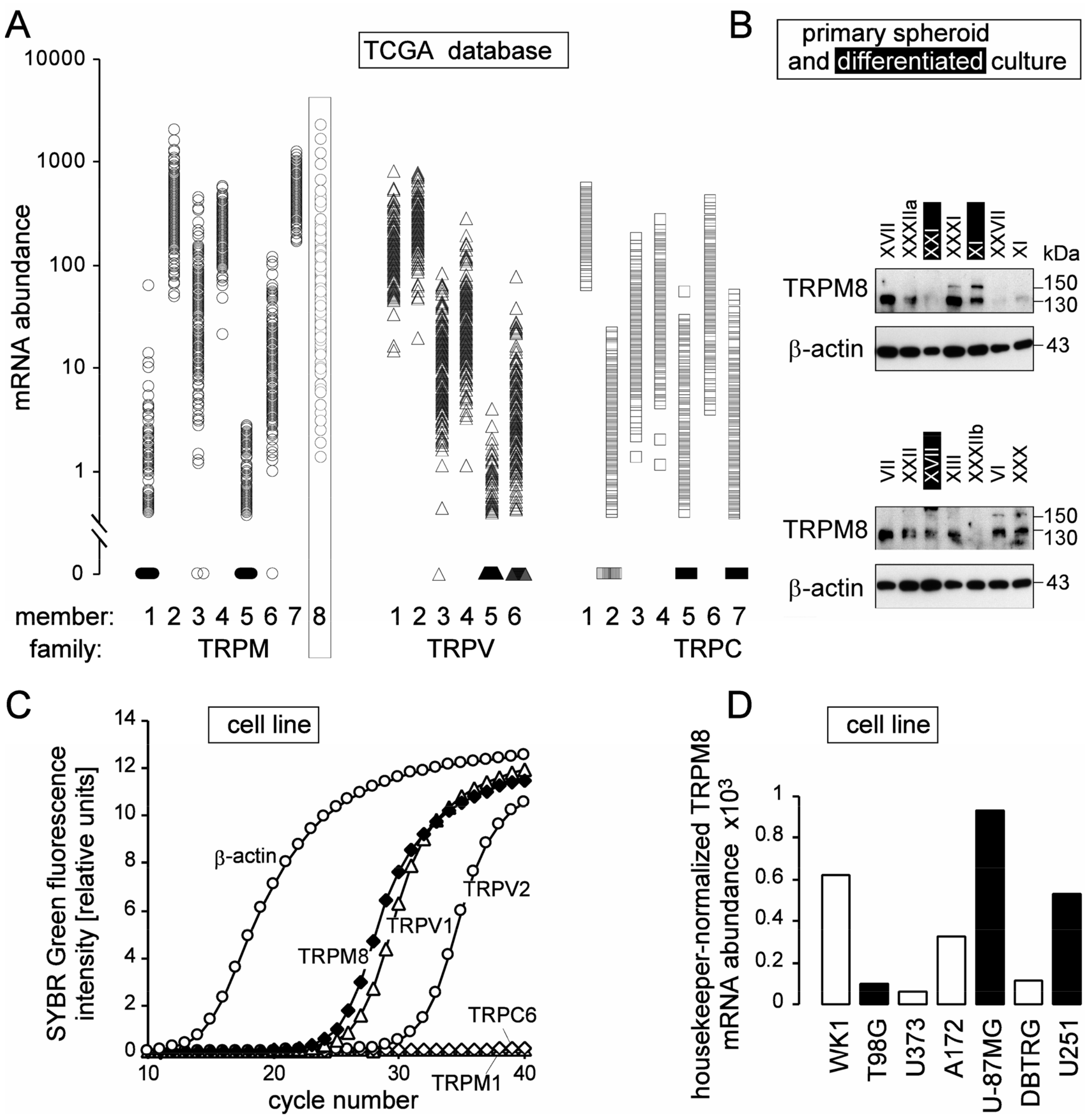

Supplementary Figure 1: TRPM8 in glioblastoma. (A) Abundances of mRNAs encoding for members of the melastatin (TRPM), vanilloid (TRPV), and canonical (TRPC) families of transient receptor potential cation channels in human glioblastoma specimens (data from the TCGA). **(B)** Immunoblots of whole cell lysates from primary glioblastoma culture grown as spheroids (Neurocult medium) or as differentiated cultures (FCS medium) probed against TRPM8protein and for loading control against β-actin (roman letters represent anonymized patient numbers). **(C)** PCR cycle-dependent increase in SYBR green fluorescence specific for β-actin, TRPM8, TRPV1, TRPV2, TRPC6 and TRPM1 in WK1 human glioblastoma cells. **(D)** Housekeeper-normalized TRPM8 mRNA abundance in the human glioma lines WK1, T98G, U373, A172, U-87MG, DBTRG, and U251.

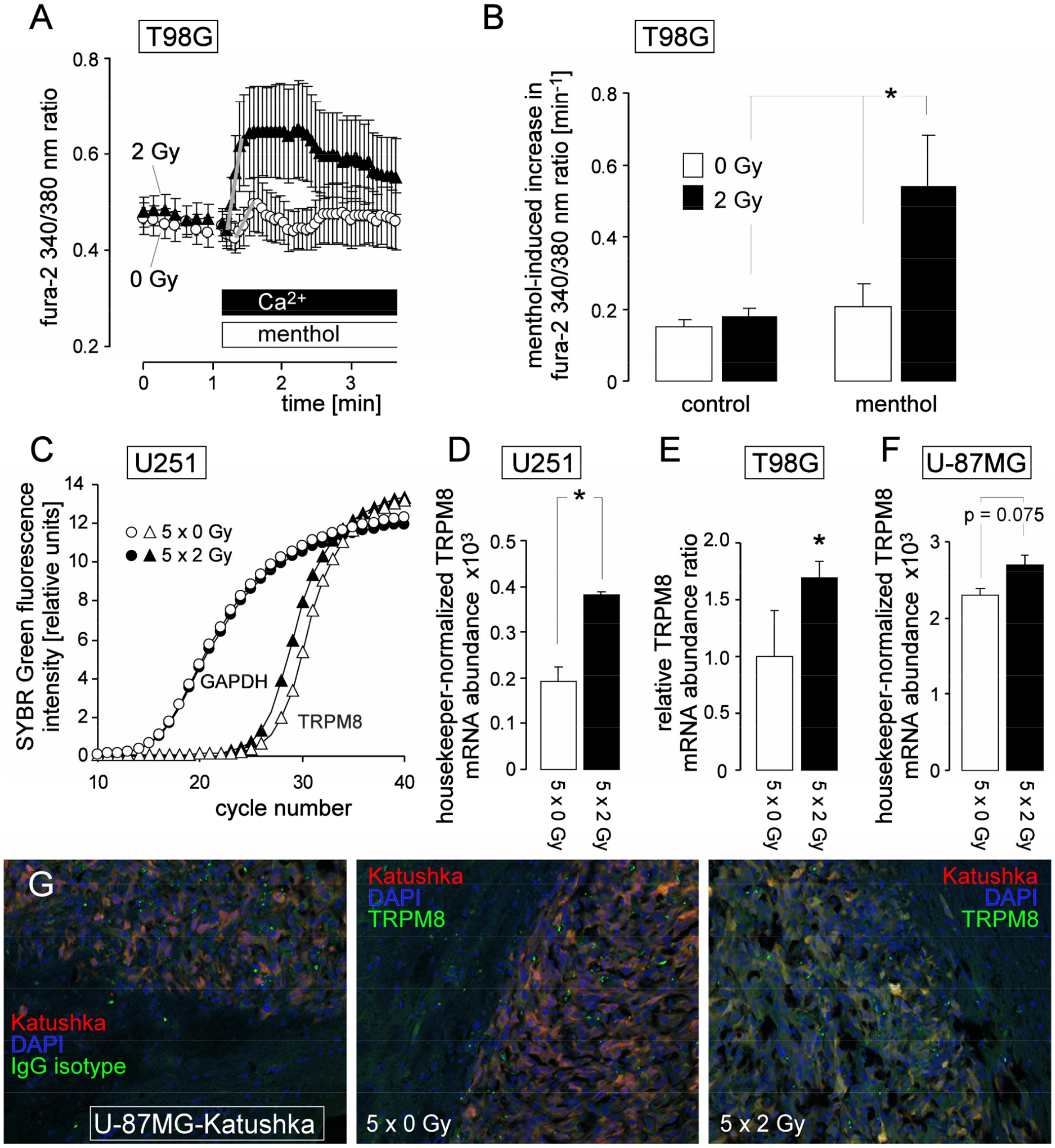

Supplementary Figure 2: Ionizing radiation induces TRPM8 activity and abundance. (A) Mean (± SE, n = 24-32) fura-2 340/380 nm fluorescence ratio as measure of cytosolic free Ca^{2+} concentration ($_{free}[Ca^{2+}]_i$) recorded in 0 Gy- (open circles) or 2 Gy-irradiated (1.5-3 h after IR, closed triangles) T98G cells. Ratios were measured during superfusion with EGTA-buffered Ca^{2+}-free NaCl solution (in mM: 125 NaCl, 32 HEPES, 5 KCl, 5 d-glucose, 1 $MgCl_2$, 0.5 EGTA, titrated with NaOH to pH 7.4) and upon wash-in of Ca^{2+} (1 mM) and menthol (100 µM). **(B)** Mean (± SE, n = 58-72) increase in $_{free}[Ca^{2+}]_i$ as determined by the slope (grey lines in A) of the rise in the 340/380 nm ratio following Ca^{2+} wash-in. Slopes were determined from 0 Gy- (open bars) or 2 Gy-irradiated T98G cells in the absence (control, left) or presence of menthol (right). **(C)** Dependence of the GAPDH- (circles) and TRPM8 (triangles) mRNA-specific SYBR green fluorescence on PCR cycle. mRNAs were extracted from cells irradiated with 5 fractions of 0 Gy (open symbols) or 2 Gy (closed symbols). **(D-F)** Mean (± SE, n = 3) GAPDH housekeeper-normalized abundance of TRPM8-specific mRNA in U251 (D), T98G (E), and (F) U-87MG cells fractionated irradiated with 5 x 0 Gy (open bars) or 5 x 2 Gy (closed bars, mRNA was extracted 24 h after the last IR fraction; in (E) GAPDH-normalized TRPM8 mRNA abundances of the irradiated cells were related to those of the respective controls (5 x 0 Gy) cells). **(G)** Fluorescence micrograph showing cryosections of mouse brain bearing human U-87MG-Katushka glioblastoma. Depicted are the margin of a tumor fractionated irradiated with 5 x 0 Gy (middle) and 5 x 2 Gy (right). Green, blue and red fluorescence indicate TRPM8-specific immunostaining, nuclear staining by DAPI, and the Katushka far-red fluorescence protein, respectively. For control, an IgG isotope (green) instead of the anti-TRPM8 antibody was applied (G, left). * indicates $p \leq 0.05$, ANOVA in (B), two-tailed (Welch-corrected) t-test in (D, F), and one-sample two-tailed t-test (significant difference from 1.0) in (E).
